# LPS-Induced Inflammatory Microenvironment Aggravates Urothelial Cell Damage and MAP Crystal Adhesion

**DOI:** 10.3390/cells15211983

**Published:** 2026-10-30

**Authors:** Jia-Yi Zhang, Xin-Yi Tong, Run-Min Tan, Zhi-Lin Yu, Jian-Ming Ouyang

**Affiliations:** College of Chemistry and Materials Science, Jinan University, Guangzhou 510632, China

**Keywords:** lipopolysaccharide, magnesium ammonium phosphate, cellular inflammation, crystal adhesion, cell damage

## Abstract

**Highlights:**

**What are the main findings?**

- OPN, ICAM-1, and CD44 participate in the adhesion process between MAP crystals and SV-HUC-1 cells.
- MAP crystals induce inflammatory responses in SV-HUC-1 cells via the ROS–CTSB–NLRP3 signaling pathway.
- The LPS-induced inflammatory microenvironment amplifies MAP crystal-induced inflammation and enhances crystal–cell adhesion.

**What are the implications of the main findings?**

- This study suggests a potential mechanism underlying MAP crystal-induced urothelial injury and increased crystal adhesion from the perspective of the inflammatory microenvironment.
- Suppression of inflammatory cascades and blockade of crystal adhesion may offer promising strategies for subsequent investigations.

**Abstract:**

Magnesium ammonium phosphate (MAP) stones feature a markedly high recurrence rate. The inflammatory microenvironment contributes to MAP crystal-induced urothelial injury, yet the specific regulatory mechanisms remain unclear. In this study, MAP crystals were synthesized, and pharmacological intervention with the ROS scavenger N-acetylcysteine (NAC), the CTSB inhibitor CA-074 Me, and the NLRP3 inhibitor MCC950 was used to explore the signaling pathway through which MAP crystals induce inflammatory responses; an inflammatory model of SV-HUC-1 urothelial cells was established using lipopolysaccharides (LPSs) to observe the regulation of key molecules in the inflammatory signaling pathway and its effects on MAP crystal adhesion and cellular injury. Results showed that MAP crystals elevated intracellular ROS levels, aggravated lysosomal membrane permeabilization, triggered CTSB leakage, and activated the NLRP3 inflammasome pathway. In the LPS-induced inflammatory microenvironment, MAP crystals exacerbated cellular inflammatory responses and cell injury. Meanwhile, the expression of adhesion-related proteins, including OPN, CD44, and ICAM-1, was further upregulated, accompanied by aggravated MAP crystal adhesion. In summary, MAP crystals induced inflammation in SV-HUC-1 cells via the ROS–CTSB–NLRP3 axis, and the LPS-induced inflammatory microenvironment further amplified cellular inflammation, cell damage, and crystal adhesion. This study provides theoretical evidence supporting the pro-inflammatory effect of the inflammatory microenvironment on MAP stone formation.

## 1. Introduction

A magnesium ammonium phosphate (MAP) stone is a typical infectious stone characterized by rapid progression and a high recurrence rate, and its formation is closely associated with urinary tract infections triggered by urease-producing bacteria [1,2]. The urease secreted by bacteria can decompose urea to alkalinize urine, which provides essential chemical conditions for MAP crystal nucleation and precipitation [3]. Biofilms formed upon bacterial attachment to the urothelium act as a crystal nucleus [3,4,5], facilitating crystal retention and growth [3,6]. In addition, bacteria detach from mature biofilms and disseminate to other urinary tract segments, where they initiate new cycles of adhesion and biofilm formation. This amplifies infectious lesions and accelerates the growth and expansion of infectious stones [7]. Apart from chemical lithogenic and biofilm effects, local inflammatory responses induced by urinary tract infection aggravate cellular injury and accelerate crystal retention and infectious stone development [8,9]. However, the specific molecular mechanisms by which inflammation drives the progression of MAP stones remain rarely reported.

Lipopolysaccharides (LPSs), an outer-membrane component of Gram-negative bacteria with endotoxin activity, can induce inflammatory response [10,11]. During urinary tract infection, colonized and proliferative bacteria can release LPSs into the urinary tract [5,6,7], which upregulates the expression of multiple inflammatory mediators, including tumor necrosis factor-α (TNF-α), interleukin-1β (IL-1β), and interleukin-6 (IL-6), eventually generating an inflammatory environment [12,13,14,15]. LPSs are abundantly enriched in infectious stone lesions. Relevant research [16] showed that LPS levels in infection stones were 36-fold higher than those in metabolic stones. LPSs secreted by *Proteus mirabilis*, the primary pathogenic bacterium, could reach concentrations as high as 14,700 μg/mL [17], and the LPS concentrations in biofilms and severely infected regions may reach even higher levels [4,6]. Most current studies construct in vitro inflammatory models via LPS stimulation; this represents a well-established experimental system for urolithiasis research [18,19]. A previous study applied LPS concentrations as high as 500 μg/mL for cellular in vitro assays [20]. Moreover, several studies have also conducted experiments using high doses of LPSs (100 or 200 μg/mL) in SV-HUC-1 cells [21,22]; immune cell lines, renal tubular epithelial cells, and urothelial cell lines are widely adopted in urolithiasis research [23,24,25]. Compared with immune cell lines such as THP-1, urothelial cell lines require substantially higher LPS concentrations to trigger a significant inflammatory response [26]. Studies [15,27,28] have shown that low concentrations of LPSs were commonly used to induce inflammation in renal tubular epithelial cells, including HK-2 cells, whereas high doses of LPS alone or combined with ATP were frequently required to establish an inflammatory model in SV-HUC-1 cells [29,30,31,32].

LPS is widely involved in the formation and progression of urinary stones. Multiple studies have shown that LPS stimulation upregulates the expression of adhesion molecules such as CD44, OPN and hyaluronic acid (HA) [33,34,35,36]. This effect strengthens crystal–cell interactions, provides favorable conditions for crystal adhesion and retention, and further facilitates stone development [37]. In calcium oxalate (CaOx) crystallization research, Kanlaya et al. [38] observed that purified LPSs effectively promoted CaOx nucleation and aggregation while markedly increasing crystal particle size. Moreover, Prywer et al. [39,40] reported that LPSs participated in the growth and aggregation behavior of phosphate calcium amorphous precipitates (PCaAPs), which are a type of infectious stone. Nevertheless, the specific mechanisms by which LPSs regulate the formation of MAP infectious stones have not yet been fully elucidated.

The NLRP3 inflammasome is one of the core signaling pathways for crystal-induced inflammation. Abnormal accumulation of reactive oxygen species (ROS) and lysosomal damage that leads to the cytosolic release of cathepsin B (CTSB) act as critical upstream events for NLRP3 inflammasome activation [41,42]. Current studies have confirmed that monosodium urate crystals [43], cholesterol crystals [44], and hydroxyapatite crystals [45] can activate the NLRP3 inflammasome pathway. Nevertheless, the specific mode by which MAP crystals modulate NLRP3 inflammasome activation remains to be further elucidated.

Based on the above research gaps, MAP crystals were synthesized in this study. An inflammatory microenvironment model of SV-HUC-1 urothelial cells was established using 250 μg/mL LPSs [46,47], and a dual-stimulation experimental model with combined LPS and MAP crystal treatment was further constructed. Compared with single stimulation, this model better recapitulates the pathological features of coexisting inflammation and crystal deposition in MAP stone lesions. Pharmacological intervention assays were performed using the ROS scavenger NAC, the CTSB inhibitor CA-074 Me, and the NLRP3 inhibitor MCC950. This study aims to elucidate the inflammatory signaling pathways triggered by MAP crystals, clarify the regulatory effect of the inflammatory microenvironment on MAP crystal adhesion and urothelial injury, and provide theoretical evidence for mechanistic research on inflammation-mediated MAP stone formation.

## 2. Materials and Methods

### 2.1. Materials and Instruments

Materials: SV-HUC-1 cells (Cellosaurus accession number: CVCL_3798) and Ham’s F-12K medium (Cat. No. PM150910), which were obtained from Procell Life Science & Technology Co., Ltd. (Wuhan, China); lipopolysaccharides (LPSs) (Cat. No. L8880), purchased from Solarbio (Beijing, China); fetal bovine serum (FBS) (Cat. No. A5256701), 0.25% trypsin–EDTA (Cat. No. 25200072), and penicillin–streptomycin (10,000 U/mL, Cat. No. 15140-122), which were supplied by Gibco (Waltham, MA, USA); cell counting kit-8 (Cat. No. 100-120, GOONIE, Guangzhou, China); CA-074 Me (Cat. No. HY-100350) and MCC950 (Cat. No. HY-12815A), which were obtained from MedChemExpress (Monmouth Junction, NJ, USA); N-acetylcysteine (NAC, Cat. No. A9165, Sigma-Aldrich, Darmstadt, Germany); universal antibody dilution buffer (Cat. No. PS119, Epizyme, Shanghai, China); 4% paraformaldehyde fixative solution (Cat. No. BL539A) and TBST washing buffer (Cat. No. BL346A), which were supplied by Biosharp (Hefei, China); OPN-neutralizing antibody (Clone MPIIIB10, Cat. No. T9901A-542) and mouse IgG1 kappa isotype control antibody (Cat. No. T78336), which were purchased from TargetMol (Boston, MA, USA); CD44-neutralizing antibody (Clone IM7, Cat. No. BE0039), which was purchased from Bio X Cell (Lebanon, NH, USA); rat IgG2b isotype control antibody (Cat. No. I-1034), which was purchased from Leinco (St. Louis, MO, USA); ICAM-1-neutralizing antibody (Clone BBIG-I1 (11C81) Cat. No. BBA3) and mouse IgG1 isotype control antibody (Cat. No. MAB002), which were purchased from R&D Systems (Minneapolis, MN, USA); ammonium dihydrogen phosphate (NH_4_H_2_PO_4_), which was purchased from Shanghai Macklin Biochemical Technology Co., Ltd. (Shanghai, China); Mg(CH_3_COO)_2_; acridine orange (AO) (Cat. No. A121748), which was purchased from Shanghai Aladdin Biochemical Technology Co., Ltd. (Shanghai, China).

Instruments: D/max 2400 X-ray powder diffractometer (Rigaku, Akishima, Tokyo, Japan); field emission scanning electron microscope (SEM) (ULTRA55, ZEISS, Oberkochen, Germany); X-ray diffractometer (XRD) (miniflex600, Rigaku, Tokyo, Japan); multi-mode microplate reader (SafireZ, Tecan, Männedorf, Switzerland); confocal laser scanning microscope (LSM 800, ZEISS, Germany); inverted fluorescence microscope (Axio Observer 3, ZEISS, Germany); multifunctional chemiluminescence imaging instrument (GE Amersham Imager 600, Cytiva, Marlborough, MA, USA); KQ3200 DE-type ultrasonic instrument (Kunshan Ultrasonic Instrument Co., Ltd., Kunshan, China).

### 2.2. Synthesis and Characterization of MAP Crystals

#### 2.2.1. Synthesis of MAP Crystals

MAP crystals were synthesized according to Reference [48]. In brief, 1.1503 g of NH_4_H_2_PO_4_ was weighed and fully dissolved, and the resulting solution was transferred into a 50 mL volumetric flask, followed by pH adjustment to 9.0 using ammonium hydroxide. Ultrapure water was added to the scale mark to prepare 50 mL of 0.2 mol/L NH_4_H_2_PO_4_ solution. Separately, 50 mL of 0.2 mol/L Mg(CH_3_COO)_2_ solution was prepared. This Mg(CH_3_COO)_2_ solution was rapidly added to the NH_4_H_2_PO_4_ solution under continuous stirring at 600 rpm at room temperature. After a 25 min reaction, the mixture was immediately subjected to suction filtration. The resulting precipitate was washed once with ultrapure water and three times with anhydrous ethanol, and the collected sample was dried prior to storage for subsequent experiments.

#### 2.2.2. Characterization of MAP Crystals

(1) Observation of morphology and size of MAP crystals by SEM.

The dried MAP crystals were mounted onto a sample holder with conductive adhesive. After gold sputter coating, the morphology and particle size of the MAP crystals were observed by SEM. The particle sizes of the crystals were measured using Image J software (Version 1.53q, National Institutes of Health, Bethesda, MD, USA), and the obtained data were further processed in Origin 2021 (Version 2021, OriginLab Corporation, Northampton, MA, USA) to fit the particle size distribution, from which the average particle size and size distribution range of the crystals were acquired.

(2) Phase composition analysis of MAP crystals by XRD.

The dried MAP crystals were ground into homogeneous powder for qualitative phase analysis via XRD. The measurement parameters were set as follows: Cu Kα radiation, an operating voltage of 40 kV, an operating current of 15 mA, a scanning range from 10° to 50°, a scanning rate of 8°/min, and a step size of 0.02°. Phase identification of the sample was performed based on the collected diffraction patterns.

### 2.3. Cell Culture and Treatments

SV-HUC-1 cells were cultured in Ham’s F-12K medium with 10% fetal bovine serum, 100 U/mL penicillin, and 100 μg/mL streptomycin in a humidified incubator at 37 °C with 5% CO_2_. Cells were digested with 0.25% trypsin–EDTA, followed by routine subculture or assignment to corresponding experimental treatments according to experimental requirements.

#### 2.3.1. Group Design for ROS, CTSB, and NLRP3 in MAP Crystal-Triggered Inflammation

To investigate the roles of ROS, CTSB and NLRP3 in MAP crystal-induced cellular inflammation, independent intervention experiments were conducted using the specific inhibitors NAC, CA-074 Me and MCC950. Four experimental groups were set up for each inhibitor intervention, namely, a control group, inhibitor group, MAP group, and MAP + inhibitor group. Cells were pretreated with each inhibitor for 2 h at working concentrations of 5 mM NAC [49,50], 10 μM CA-074 Me [45,51], and 10 μM MCC950 [52,53]. After pretreatment, MAP crystals were supplemented to reach a final concentration of 1.0 mg/mL for additional 12 h incubation.

#### 2.3.2. Group Design for Cellular Injury and Adhesion by MAP Crystals

To explore the influence of the inflammatory microenvironment on MAP crystal-induced cellular injury and adhesion, four experimental groups were set up, namely, a control group, LPS group, MAP group, and LPS + MAP group. The treatment timeline was as follows: 0–12 h: culture medium of cells in all groups was replaced with serum-free medium, followed by pretreatment with 250 μg/mL LPSs (an equal volume of vehicle was added for the control group). LPSs were removed by PBS washing after pretreatment. At 12–24 h: cells were stimulated with 1.0 mg/mL MAP crystals (an equal volume of vehicle was added for the control group). Samples were harvested at 24 h.

#### 2.3.3. Group Design for OPN-, CD44-, and ICAM-1-Mediated MAP Crystal–Cell Interactions

To verify the mediating effects of the adhesion molecules OPN, CD44 and ICAM-1 in the interaction between MAP crystals and cells, neutralizing-antibody-blocking experiments were performed. Neutralizing antibodies were used to specifically block target membrane proteins and inhibit crystal–cell binding. The experimental groups were designed as follows [54,55,56]: a control group, MAP group, MAP + anti-OPN group, MAP + OPN-IgG group, MAP + anti-CD44 group, MAP + CD44-IgG group, MAP + anti-ICAM-1 group, and MAP + ICAM-1-IgG group. Cells were pretreated for 2 h with each neutralizing antibody or corresponding isotype-matched IgG control at the following working concentrations: amounts of 4 μg/mL for anti-OPN and OPN-IgG; 10 μg/mL for anti-CD44 and CD44-IgG; 10 μg/mL for anti-ICAM-1 and ICAM-1-IgG. After pretreatment, the control group was maintained in serum-free medium, while the other groups were incubated with MAP crystals at a final concentration of 1.0 mg/mL for 12 h.

### 2.4. Evaluation of Cell Viability with CCK-8 Assay

Group settings are presented in Section 2.3.2. Cells were seeded into 96-well plates with three replicate wells for each experimental group. After completion of the designated incubation, the culture medium in the wells was discarded. Subsequently, 100 μL of serum-free medium containing 10% CCK-8 working solution was added to each well, followed by incubation in the dark at 37 °C for 1.5 h. The optical density (OD) value at 450 nm of each well was measured using an enzyme detector (SafireZ, Tecan, Switzerland). The formula for calculating relative cell viability is as follows:

Relative cell viability (%) = [OD(damaged group) − OD(blank group)]/[OD(control group) − OD(blank group)] × 100%(1)

### 2.5. Intracellular ROS Detection by Confocal Microscopy

Group settings are presented in Section 2.3.1 and Section 2.3.2. The group conditions in Section 2.3.1 were used to explore the effect of NAC inhibition on MAP crystal-induced ROS production, while those in Section 2.3.2 were used to assess ROS generation under the LPS-induced inflammatory microenvironment. Intracellular ROS levels were detected according to the protocol of the manufacturer of the ROS assay kit (Cat. No. S0036S, Beyotime, Haimen, Nantong, China). After completion of the designated incubation, the culture medium was removed, and cells were washed three times with PBS. A CM-H_2_DCFDA fluorescent probe was diluted at 1:1000 with probe dilution buffer to obtain the staining solution. The staining solution was then added to fully cover the cells. Cells were incubated in a humidified CO_2_ incubator at 37 °C for 30 min, followed by three PBS washes to remove any extracellular CM-H_2_DCFDA.

Fluorescence images were captured using a confocal microscope (LSM 800, ZEISS, Germany) [57], and the green fluorescent signal of CM-H_2_DCFDA was acquired by the FITC channel. Image resolution was set to 1024 × 1024 pixels. Laser power, gain and offset were optimized at initial setup and kept constant across all experimental groups. Fluorescence images were analyzed using Image J software (Version 1.53q) [57,58]. Identical threshold and analytical parameters were applied to measure the mean intracellular ROS fluorescence intensity in each group. Final results are presented as fold changes relative to the control group.

### 2.6. Analysis of CTSB, NLRP3, Cleaved Caspase-1 (p20), IL-1β, IL-18, OPN, and ICAM-1 Protein Expression by Western Blot

Group settings are presented in Section 2.3.1 and Section 2.3.2. The group conditions in Section 2.3.1 were used to explore the regulatory effects of inhibitor treatment on MAP crystal-induced cellular inflammatory responses, while those in Section 2.3.2 were used to explore the influence of the inflammatory microenvironment on MAP crystal-induced cellular injury and adhesion. Western blotting was performed according to previously described protocols [45,46]. After completion of the designated incubation, cells were washed three times with pre-chilled PBS and lysed on ice in RIPA lysis buffer (Cat. No. P0013B, Beyotime, China) supplemented with a protease inhibitor cocktail (Cat. No. CW2200S, CWBIO, Taizhou, China) and a phosphatase inhibitor cocktail (Cat. No. P1081, Beyotime, China). Cell lysates were centrifuged at 12,000 rpm for 15 min at 4 °C to remove cellular debris. The total protein concentration of the resulting supernatant was determined using a BCA protein assay kit (Cat. No. CW0014S, CWBIO, China). SDS-containing loading buffer was added to the protein supernatant, and all samples were adjusted to identical protein concentrations. The samples were then denatured at 100 °C for 10 min. After cooling, the denatured protein samples were preserved for subsequent experiments.

Proteins were separated by sodium dodecyl sulfate–polyacrylamide gel electrophoresis (SDS-PAGE) and subsequently wet-transferred onto nitrocellulose (NC) membranes at 200 mA for 90 min at 4 °C. After washing with TBST, membranes were blocked with 5% skimmed milk for 2 h at room temperature. Following another TBST wash, membranes were incubated with the corresponding primary antibody at 4 °C overnight with gentle shaking. The primary antibodies used were as follows: CTSB (1:3000, Cat. No. 12216-1-AP, Proteintech, Wuhan, China); NLRP3 (1:7000, Cat. No. 68102-1-Ig, Proteintech, China); Cleaved Caspase-1 (p20) (1:3500, Cat. No. 22915-1-AP, Proteintech, China); IL-1β (1:5000, Cat. No. 16806-1-AP, Proteintech, China); IL-18 (1:20,000, Cat. No. 10663-1-AP, Proteintech, China); OPN (1:1000, Cat. No. 22952-1-AP, Proteintech, China); ICAM-1 (1:20,000, Cat. No. 60299-1-Ig, Proteintech, China); and α-Tubulin (1:50,000, Cat. No. 66031-1-Ig, Proteintech, China).

On the next day, membranes were thoroughly washed with TBST and incubated with HRP-conjugated goat anti-mouse secondary antibody (1:10,000, Cat. No. bs-0296G-HRP, Bioss, Beijing, China) or goat anti-rabbit secondary antibody (1:20,000, Cat. No. SA00001-2, Proteintech, China) for 2 h at room temperature. Protein bands were visualized using an ECL chemiluminescence substrate (Cat. No. PMK003, BIOPRIMACY, Wuhan, China), and images were acquired using a multifunctional chemiluminescence imaging system (GE Amersham Imager 600, Cytiva, Marlborough, MA, USA). Band gray values were quantified using Image J software (Version 1.53q). The intensity of each target band was normalized to α-Tubulin. Data are presented as percentages relative to the control group. Uncropped original Western blot images are provided in Supplementary Figures S1–S6.

### 2.7. Quantification of IL-18, IL-1β, IL-6 and TNF-α by Enzyme-Linked Immunosorbent Assay (ELISA)

Group settings are presented in Section 2.3.1. Secretion levels of IL-18, IL-1β, IL-6 and TNF-α were measured by ELISA. Commercial ELISA kits were used for detection of cytokines, including IL-18 (Cat. No. E-EL-H0253, Elabscience, Wuhan, China), IL-1β (Cat. No. E-EL-H0149, Elabscience, China), IL-6 (Cat. No. S201604, DiBai Biotechnology, Shanghai, China) and TNF-α (Cat. No. SEKH-0047, Solarbio, China). All experimental procedures were performed strictly in accordance with the manufacturer’s instructions. Before the experiment, the kits were balanced to room temperature. Briefly, the experimental workflow involved loading standards and samples, incubation with biotinylated detection antibodies, incubation with HRP conjugate working solution, substrate color development in the dark, and reaction termination. The OD values at 450 nm were measured using an enzyme detector (SafireZ, Tecan, Switzerland), and the concentrations were calculated based on the standard curve.

### 2.8. Determination of Cell Death by LDH Quantification

Group settings are presented in Section 2.3.1. According to the manufacturer’s protocol for the LDH cytotoxicity assay kit (Cat. No. C0017, Beyotime, China), additional groups, including a background blank control group and maximum enzyme activity well group, were established. After completion of the designated incubation, culture plates were centrifuged at 400× *g* for 5 min. Subsequently, 120 μL of supernatant from each well was transferred to the corresponding wells of a new 96-well plate. Prior to detection, the LDH substrate solution, 1 × INT solution and enzyme solution were mixed at equal volumes to prepare the LDH working solution. Subsequently, 60 μL of LDH working solution was added to each well, followed by thorough mixing and incubation at room temperature in the dark for 30 min. After incubation, the OD values of each well were measured at 490 nm using an enzyme detector, and the OD values of the background blank control well were subtracted from the OD values of each group. The LDH release rate was calculated using the following formula:

LDH release rate (%) = [OD(treatment group) − OD(control group)]/[OD(max group) − OD(control group)] × 100%(2)

### 2.9. Detection of Lysosomal Integrity by Confocal Microscopy

Group settings are presented in Section 2.3.2. Lysosomal integrity was detected by AO staining (Cat. No. A121748, Aladdin, Shanghai, China) [59]. Cells were seeded into confocal dishes. After completion of the designated incubation, the culture medium was discarded, and cells were washed three times with PBS. Cells were incubated with 5 μg/mL AO staining solution in a humidified incubator in the dark for 15 min. After incubation, the staining solution was discarded, and cells were washed thoroughly three times with PBS to wash away unbound dyes. AO serves as a specific indicator of lysosomal integrity. In intact lysosomes, AO exists as protonated aggregates and emits red fluorescence upon excitation. When lysosomal membrane integrity is compromised and permeability increases, AO leaks to the cytosol and exists as deprotonated monomeric, predominantly exhibiting green fluorescence. Relative changes between red and green fluorescence signals can directly reflect the degree of lysosomal damage.

Fluorescence images were acquired using a confocal microscope (LSM 800, ZEISS, Germany) [57]. Green fluorescence signals were acquired via the FITC channel, and red fluorescence signals were acquired via the Alexa Fluor 594 channel. Images with a resolution of 1024 × 1024 pixels were acquired in frame scanning mode. Laser power, gain and offset were optimized at initial setup and kept constant across all experimental groups. Relative quantitative analysis of the fluorescence images was performed using Image J software (Version 1.53q) [57,58]. Identical threshold and analytical parameters were applied to measure the mean red and green fluorescence intensities. The lysosomal integrity was evaluated based on the ratio of the mean green fluorescence to mean red fluorescence intensity. Final results are presented as percentages relative to the control group.

### 2.10. Immunofluorescence Analysis of CTSB, NLRP3, IL-1β, IL-18, TNF-α, ZO-1, and CD44 Expression

Group settings are presented in Section 2.3.2. Immunofluorescence staining was performed to detect the expression of CTSB, NLRP3, IL-1β, IL-18, TNF-α, ZO-1 and CD44 according to the protocols described in References [45,46]. Cells were seeded onto sterile 14 mm circular glass. After completion of the designated incubation, the culture medium was removed, and cells were washed three times with PBS. Cells were fixed with 4% paraformaldehyde (Cat. No. BL539A, Biosharp, China) at room temperature for 15 min. After PBS washing, cells were permeabilized with 0.1% Triton X-100 (Cat. No. BL934, Biosharp, China) for 10 min and washed three additional times with PBS. Non-specific binding sites were blocked by 5% BSA (Cat. No. A7030, Sigma-Aldrich, St. Louis, MO, USA) at room temperature for 1 h. Following removal of the blocking solution and PBS washing, cells were incubated with the corresponding primary antibody at 4 °C overnight. Primary antibody information is listed as follows: CTSB (1:500, Cat. No. 12216-1-AP, Proteintech, China); NLRP3 (1:300, Cat. No. 68102-1-Ig, Proteintech, China); IL-1β (1:300, Cat. No. 16806-1-AP, Proteintech, China); IL-18 (1:300, Cat. No. 10663-1-AP, Proteintech, China); TNF-α (1:300, Cat. No. 60291-1-Ig, Proteintech, China); ZO-1 (1:1000, Cat. No. 21773-1-AP, Proteintech, China); CD44 (1:300, Cat. No. OM642238, Omnimabs, Alhambra, CA, USA).

On the next day, cells were washed three times with PBS and incubated with Alexa Fluor^®^ 488 (1:600, Cat. No. ab150077, Abcam, Cambridge, UK) or Alexa Fluor^®^ 594 (1:600, Cat. No. ab150080, Abcam, UK) secondary antibodies at room temperature in the dark for 1 h. Following PBS washes, coverslips were mounted using anti-fade mounting medium containing DAPI (Cat. No. P0131, Beyotime, China) for nuclear counterstaining, and the edges of the coverslips were sealed.

Fluorescence images were acquired using a confocal microscope (LSM 800, ZEISS, Germany) [57]. Nuclear signals were acquired via the DAPI channel, and target protein fluorescence signals were acquired via the Alexa Fluor 488 or Alexa Fluor 594 channel. Images with a resolution of 1024 × 1024 pixels were acquired in frame scanning mode. Laser power, gain and offset were optimized at initial setup and kept constant across all experimental groups. Relative quantitative analysis of the fluorescence images was performed using Image J software (Version 1.53q) [58,59]. Identical threshold and analytical parameters were applied to measure the mean fluorescence intensity of the target proteins in each group. Final results are expressed as fold changes relative to the control group.

### 2.11. Observation of Cytoskeletal F-Actin by Phalloidin Staining

Group settings are presented in Section 2.3.2. The F-actin cytoskeleton was stained using an F-actin fluorescent probe (Cat. No. C2201S, Beyotime, China) according to the manufacturer’s instructions. Cells were seeded onto sterile 14 mm glass. After completion of the designated incubation, the culture medium was removed, and cells were washed three times with PBS. Cells were fixed with 4% paraformaldehyde at room temperature for 15 min, followed by two washes with PBS containing 0.1% Triton X-100 for 5 min each. The F-actin staining working solution was prepared by diluting the stock probe at a volume ratio of 1:100 using immunofluorescence secondary antibody dilution buffer (Cat. No. P0108, Beyotime, China). An adequate volume of working solution was added to fully cover the cell samples, and samples were incubated at room temperature in the dark for 45 min. After incubation, cells were washed three times with PBS containing 0.1% Triton X-100. Coverslips were mounted using anti-fade mounting medium containing DAPI for nuclear staining, and the edges of the coverslips were sealed.

Fluorescence images were acquired using a laser scanning confocal microscope (LSM 800, ZEISS, Germany) [57]. Nuclear signals were acquired via the DAPI channel, and green F-actin fluorescence signals were acquired via the FITC channel. Images with a resolution of 1024 × 1024 pixels were acquired in frame scanning mode. Laser power, gain and offset were optimized at initial setup and kept constant across all experimental groups.

### 2.12. Observation of MAP Cell Membrane Morphology and Crystal–Cell Adhesion by SEM

Group settings are presented in Section 2.3.2. Cells were seeded onto sterile 14 mm diameter glass. After completion of the designated incubation, the culture medium was removed, and cells were washed three times with PBS to remove unbound MAP crystals. Subsequently, cells were fixed with an electron microscopy fixative (Cat. No. G1102, Servicebio, Wuhan, China) at 4 °C for 30 min. After discarding the fixative, cells were rinsed three times with PBS and subjected to serial dehydration in a graded ethanol series (30%, 50%, 70%, 80%, 90%, 100%). Subsequently, specimens were dried via CO_2_ critical-point drying and sputter-coated with gold. MAP crystal–cell adhesion and cell membrane morphology were observed using an SEM (ULTRA55, ZEISS, Germany).

### 2.13. Observation of MAP Crystal–Cell Adhesion by Inverted Fluorescence Microscope

Group settings are presented in Section 2.3.2 and Section 2.3.3. The group conditions in Section 2.3.2 were used to explore the influence of the inflammatory microenvironment on MAP crystal-induced cellular adhesion, while those in Section 2.3.3 were used to verify the mediating effects of the adhesion molecules OPN, CD44 and ICAM-1 in the interaction between MAP crystals and cells. Cells were seeded into 12-well plates. After completion of the designated incubation, the supernatant was removed, and cells were washed three times with PBS to wash away unbound MAP crystals. Bright-field images were acquired using an inverted fluorescence microscope (Axio Observer 3, ZEISS, Germany). At least five randomly selected fields of view were imaged for each experimental group. Semi-quantitative analysis of crystal adhesion was performed using Image J software (Version 1.53q). An identical threshold was applied to all images to automatically segment cellular regions and crystal particles. The crystal adhesion index was defined as the number of adherent crystals per unit cell area to quantitatively assess MAP crystal–cell adhesion across experimental groups.

### 2.14. Statistical Analysis

Statistical analyses were carried out using GraphPad Prism software (Version 10.1.2, GraphPad Software, San Diego, CA, USA). All data are presented as mean ± standard deviation (Mean ± SD). Each experiment included at least three independent biological replicates (n ≥ 3), and the exact n for each experiment is indicated in the corresponding figure legends. Each biological replicate was derived from an independently cultured batch of cells. Technical replicates (e.g., duplicate wells or triplicate measurements) were averaged to obtain a single value for each biological replicate. Formal normality testing was not performed because the small sample size in this study was insufficient to reliably assess data normality. Two-way ANOVA was applied for the 2 × 2 factorial design, with Tukey’s post hoc pairwise comparisons performed when a significant effect was detected. One-way ANOVA with Tukey’s post hoc test was applied for multiple-group comparisons. Since the normality assumption could not be fully validated, the resulting *p* values should be interpreted cautiously together with individual data points, as well as the magnitude and direction of observed differences. Differences with *p* < 0.05 were regarded as statistically significant.

## 3. Results

### 3.1. Characterization of the Synthesized MAP Crystals

**Figure 1:**
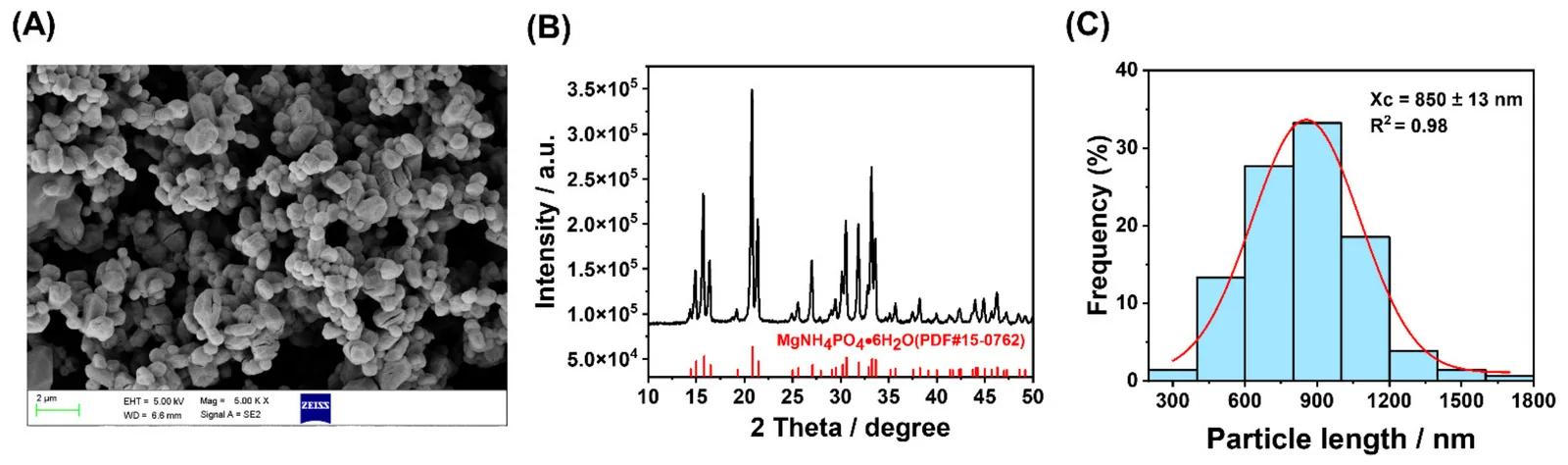
Synthesis and characterization of MAP crystals: (**A**) SEM image; (**B**) XRD spectra; (**C**) particle size distribution graph.

As shown in Figure 1A, the synthesized MAP crystals exhibited smooth, flat surfaces, a uniform size, and an ellipsoidal morphology. The size of the crystals was measured using Image J software and averaged 850 ± 13 nm (Figure 1C).

XRD analysis (Figure 1B) indicated that the diffraction peaks of the synthesized crystals matched the standard PDF card of orthorhombic magnesium ammonium phosphate hexahydrate (NH_4_MgPO_4_·6H_2_O) (PDF#15-0762), and no additional impurity diffraction peak was detected, indicating that the sample contained pure-phase MAP crystals. The sharp diffraction peaks and flat baseline indicated the high crystallinity of the sample.

### 3.2. Regulatory Effects of NAC, CA-074 Me and MCC950 on MAP Crystal-Induced Cellular Inflammatory Responses

**Figure 2:**
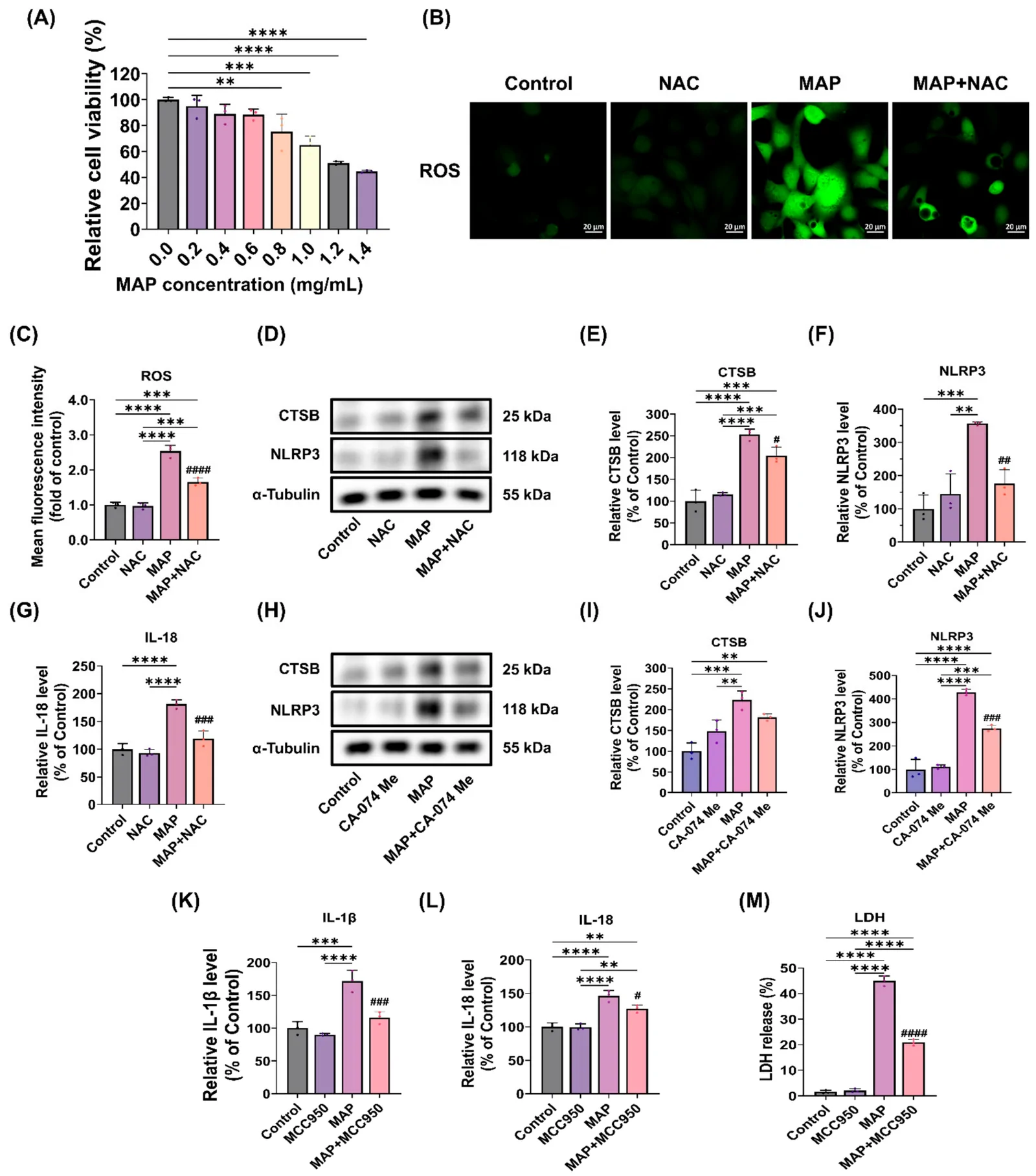
MAP crystals induce inflammatory response in SV-HUC-1 cells. (**A**) Evaluation of cell viability with CCK-8 assay. (**B**–**G**) Results from NAC intervention experiment: representative images of intracellular ROS levels (**B**), semi-quantitative analysis of ROS fluorescence intensity (**C**), representative Western blot images of CTSB and NLRP3 (**D**), semi-quantitative analysis of CTSB protein (**E**) and NLRP3 protein (**F**) based on Western blot results in (**D**), quantification of IL-18 by ELISA (**G**). Scale bar: 20 μm. (**H**–**J**) Results from CA-074 Me intervention experiment: representative Western blot images of CTSB and NLRP3 (**H**), semi-quantitative analysis of CTSB protein (**I**) and NLRP3 protein (**J**) based on Western blot results in (**H**). (**K**–**M**) Results from MCC950 intervention experiment: quantification of IL-1β (**K**) and IL-18 (**L**) by ELISA and LDH release levels (**M**). Data are presented as Mean ± SD (n = 3 biologically independent experiments). The inter-group comparisons were conducted using one-way ANOVA, followed by Tukey’s post hoc test; ** *p* < 0.01, *** *p* < 0.001, and **** *p* < 0.0001 between indicated groups; # *p* < 0.05, ## *p* < 0.01, ### *p* < 0.001, and #### *p* < 0.0001 versus MAP group.

Cell viability was detected using the CCK-8 assay after treatment with MAP crystals at serially diluted concentrations ranging from 0 to 1.4 mg/mL for 12 h. As shown in Figure 2A, MAP crystals significantly reduced the viability of SV-HUC-1 cells in a concentration-dependent manner. To sustain optimal cellular status and appropriate cell viability for subsequent experiments [60,61], 1.0 mg/mL MAP crystals were selected as the treatment concentration for subsequent detections in this study.

To preliminarily verify whether MAP crystals induce inflammatory responses in urothelial cells via the ROS–CTSB–NLRP3 pathway, pharmacological intervention with the ROS scavenger NAC, the CTSB inhibitor CA-074 Me, and the NLRP3 inhibitor MCC950 was applied to confirm the participation of each molecule within this signaling pathway.

As shown in Figure 2B,C, the intracellular ROS level in the MAP group was approximately 1.5-fold higher than that in the MAP + NAC group (*p* < 0.0001), suggesting that NAC pretreatment effectively reduced MAP crystal-induced intracellular ROS accumulation. Western blot analysis (Figure 2D–F) showed that the protein levels of CTSB and NLRP3 in the MAP group were approximately 1.2-fold and 2.0-fold higher than those in the MAP + NAC group (*p* values of 0.0365 and 0.0035, respectively), suggesting that NAC pretreatment could effectively inhibit the MAP crystal-induced expression of CTSB and NLRP3. Quantitative ELISA results (Figure 2G) showed that the IL-18 secretion level in the MAP group was approximately 1.5-fold higher than that in the MAP + NAC group (*p* = 0.0003). This finding suggested that NAC pretreatment could effectively suppress IL-18 secretion induced by MAP crystals. The above results suggested that MAP crystal-induced intracellular ROS accumulation participated in mediating lysosomal CTSB release into the cytoplasm and subsequent NLRP3 inflammasome activation.

Western blot results (Figure 2H–J) showed that the protein levels of CTSB and NLRP3 in the MAP group were approximately 1.2-fold and 1.6-fold higher than those in the MAP + CA-074 Me group after the pharmacological blockade of CTSB activity (*p* values of 0.1398 and 0.0002, respectively). These data indicated that suppressing CTSB activity reduced NLRP3 expression, suggesting that CTSB may contribute to the activation of the NLRP3 inflammatory signaling pathway.

To further verify the regulatory role of NLRP3 inflammasome activation in MAP crystal-induced urothelial inflammation, the specific NLRP3 inflammasome inhibitor MCC950 was employed in this study. Quantitative ELISA results (Figure 2K,L) suggested that the secretion levels of IL-1β and IL-18 in the culture supernatant from the MAP group were approximately 1.5-fold and 1.1-fold higher than those in the MAP + MCC950 group (*p* values of 0.0010 and 0.0255, respectively). LDH release assays further demonstrated that the LDH release level in the MAP group was approximately 2.2-fold higher than that in the MAP + MCC950 group (*p* < 0.0001). These findings indicated that the pharmacological blockade of the NLRP3 inflammasome by MCC950 markedly restrained the MAP crystal-induced release of IL-1β, IL-18 and LDH, suggesting that the NLRP3 inflammasome played an important regulatory role in the cellular inflammatory response induced by MAP crystals.

### 3.3. Establishment of LPS-Induced Inflammatory Microenvironment

**Figure 3:**
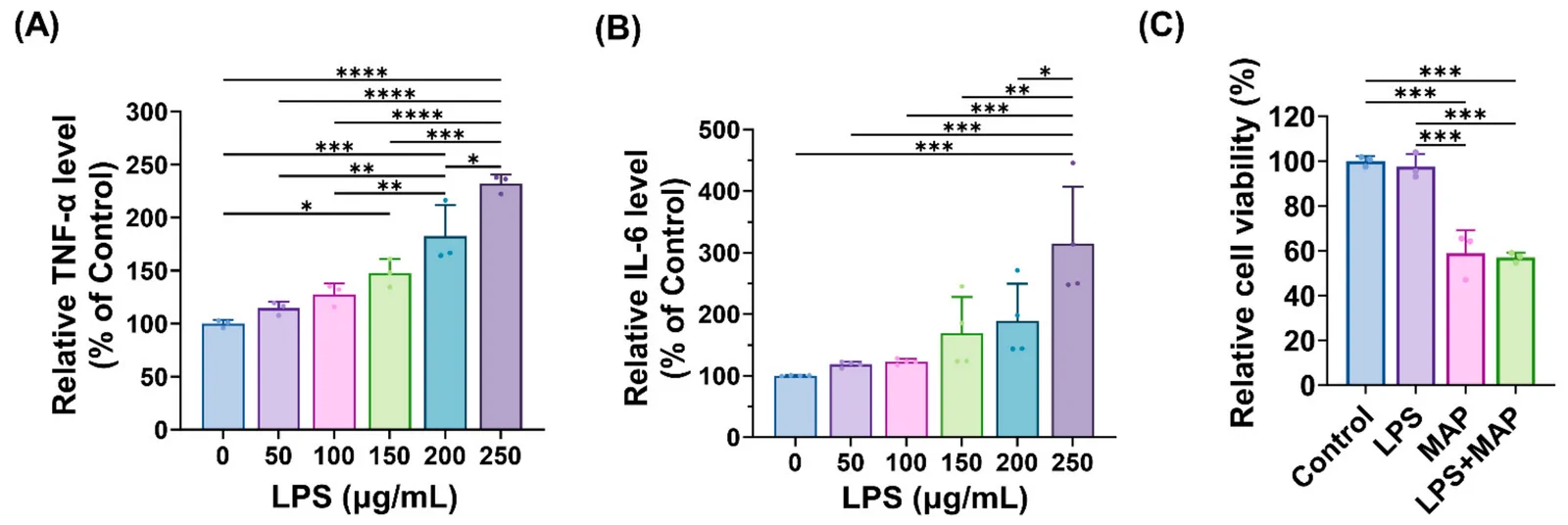
LPS-induced inflammation in SV-HUC-1 cells in vitro: (**A**) Quantification of TNF-α by ELISA (n = 3 independent biological experiments); (**B**) quantification of IL-6 by ELISA (n = 4 independent biological experiments); (**C**) evaluation of cell viability with CCK-8 assay (n = 3 independent biological experiments). Data are presented as Mean ± SD. Two-way ANOVA was applied for 2 × 2 factorial design, with Tukey’s post hoc pairwise comparisons performed. One-way ANOVA with Tukey’s post hoc test was applied for multiple-group comparisons; * *p* < 0.05, ** *p* < 0.01, *** *p* < 0.001, and **** *p* < 0.0001 between indicated groups.

The inflammatory model of urothelial SV-HUC-1 cells induced by LPSs was evaluated by detecting the expression levels of the inflammatory cytokines TNF-α and IL-6 [19,62,63,64]. Referring to the LPS concentration gradients and modeling conditions described in previous studies [21,65], cells were treated with serially diluted LPSs at concentrations ranging from 0 to 250 μg/mL for 12 h. As shown in Figure 3A,B, the TNF-α and IL-6 expression levels were closely related to the LPS concentration. At an LPS concentration of 250 μg/mL, the expression levels of TNF-α and IL-6 were approximately 2.32-fold and 3.14-fold higher than those in the control group, respectively. This concentration induced a robust cellular inflammatory response (*p* < 0.001). Therefore, 250 μg/mL LPSs was chosen as the condition for inflammatory model establishment in subsequent experiments.

As presented in Figure 3C, cell viability exhibited no significant difference between the LPS group and the control group (*p* = 0.9519), indicating that LPSs at this concentration successfully induced the cellular inflammatory model and maintained the cell survival rate. Following treatment with 1.0 mg/mL MAP crystals, the cell viability decreased to approximately 60% in both the MAP group and LPS + MAP group, and no significant difference was observed between the two groups (*p* = 0.9813). At this concentration, cells maintained an appropriate survival rate, and non-specific interference arising from differences in cell viability could be effectively eliminated, meeting the requirements of the subsequent inflammatory detection and crystal adhesion assays.

### 3.4. Effects of Inflammatory Microenvironment on MAP Crystal-Induced Subcellular Structural Damage

**Figure 4:**
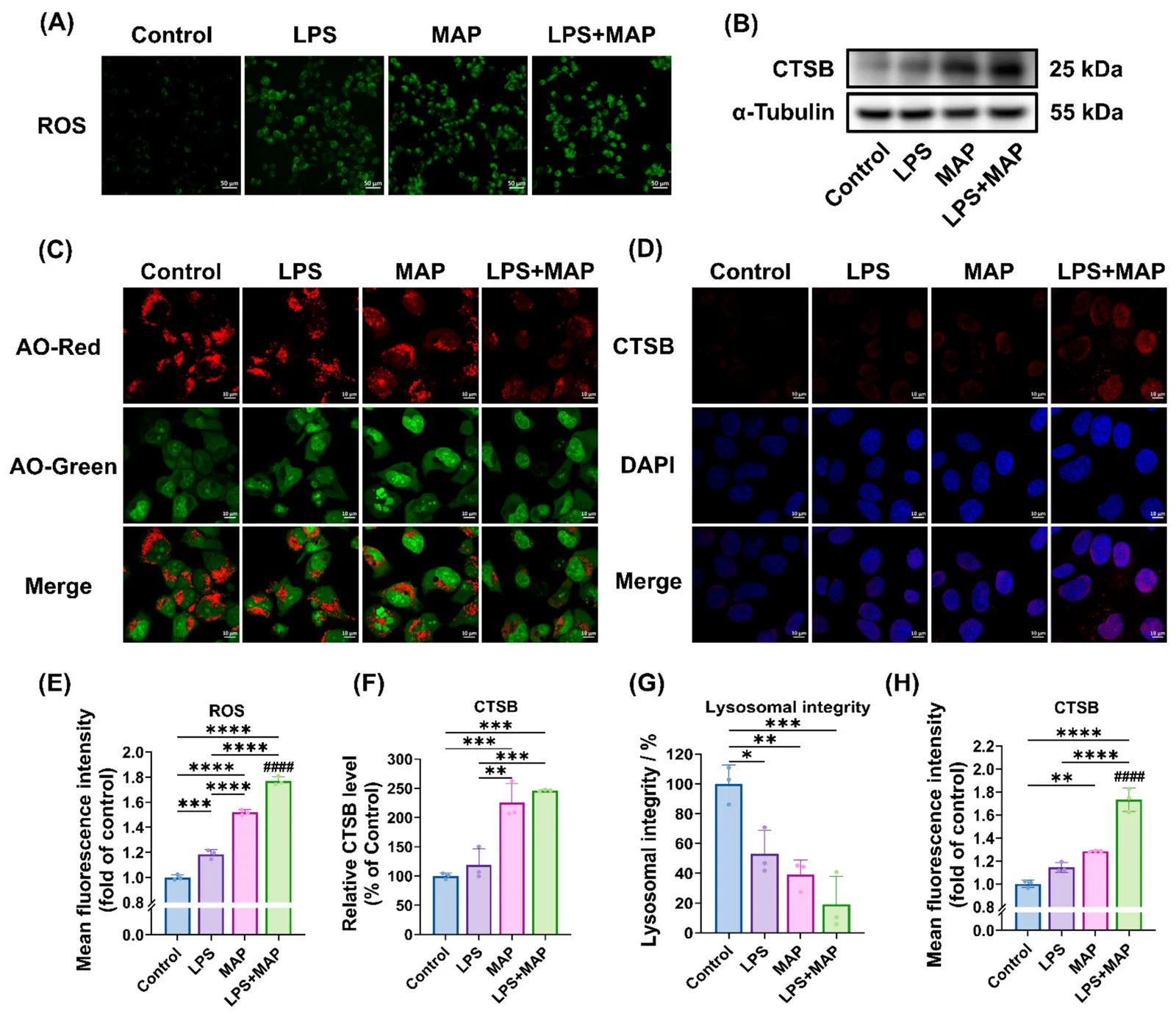
Damaging effect of MAP crystals on subcellular structures. (**A**,**E**) Representative fluorescence images (**A**) and semi-quantitative analysis (**E**) of intracellular ROS levels. Scale bar: 50 μm. (**B**,**F**) Representative Western blot images (**B**) and corresponding semi-quantitative analysis (**F**) of CTSB protein. (**C**,**G**) Representative fluorescence images (**C**) and semi-quantitative analysis (**G**) of lysosomal integrity. Scale bar: 10 μm. (**D**,**H**) Representative immunofluorescence images (**D**) and corresponding semi-quantitative analysis (**H**) of CTSB. Scale bar: 10 μm. Data are presented as Mean ± SD (n = 3 independent biological experiments). Two-way ANOVA was applied for the 2 × 2 factorial design, with Tukey’s post hoc pairwise comparisons performed; * *p* < 0.05, ** *p* < 0.01, *** *p* < 0.001, and **** *p* < 0.0001 between indicated groups; #### *p* < 0.0001 versus MAP group.

To investigate how the LPS-induced inflammatory microenvironment aggravates subcellular structure damage induced by MAP crystals, the intracellular ROS level, lysosomal integrity, and cytoplasmic CTSB content were examined in this study. As shown in Figure 4A,E, the ROS level in the LPS + MAP group was higher than those in the MAP group and LPS groups and was approximately 1.2-fold higher than that in the MAP group. AO staining (Figure 4C,G) revealed more severe lysosomal injury in the LPS + MAP group than that in the MAP group and LPS group, with the degree of lysosomal damage being approximately 2.0-fold greater than that in the MAP group. In addition, both Western blot results (Figure 4B,F) and immunofluorescence staining (Figure 4D,H) showed that cytosolic CTSB levels were higher in the LPS + MAP group than those in the MAP group or LPS group. Semi-quantitative analysis demonstrated that the cytosolic CTSB protein levels in the LPS + MAP group were approximately 1.1-fold higher than those in the MAP group based on Western blot analysis, while the immunofluorescence signal intensity was approximately 1.3-fold higher than that in the MAP group. These results indicated that the LPS-induced inflammatory microenvironment further promoted intracellular ROS levels, exacerbated lysosomal damage, and facilitated subsequent cytosolic CTSB release.

### 3.5. Effects of Inflammatory Microenvironment on MAP Crystal-Induced Cellular Inflammation

NLRP3 mediates the inflammatory response by activating cysteine-requiring aspartate-specific protease-1 (caspase-1) to facilitate the secretion of the inflammatory cytokines IL-1β and IL-18 [66,67]. The massive accumulation of inflammatory cytokines such as TNF-α, IL-1β and IL-18 may disrupt cellular homeostasis and contribute to the initiation and progression of inflammatory disorders [68,69].

**Figure 5:**
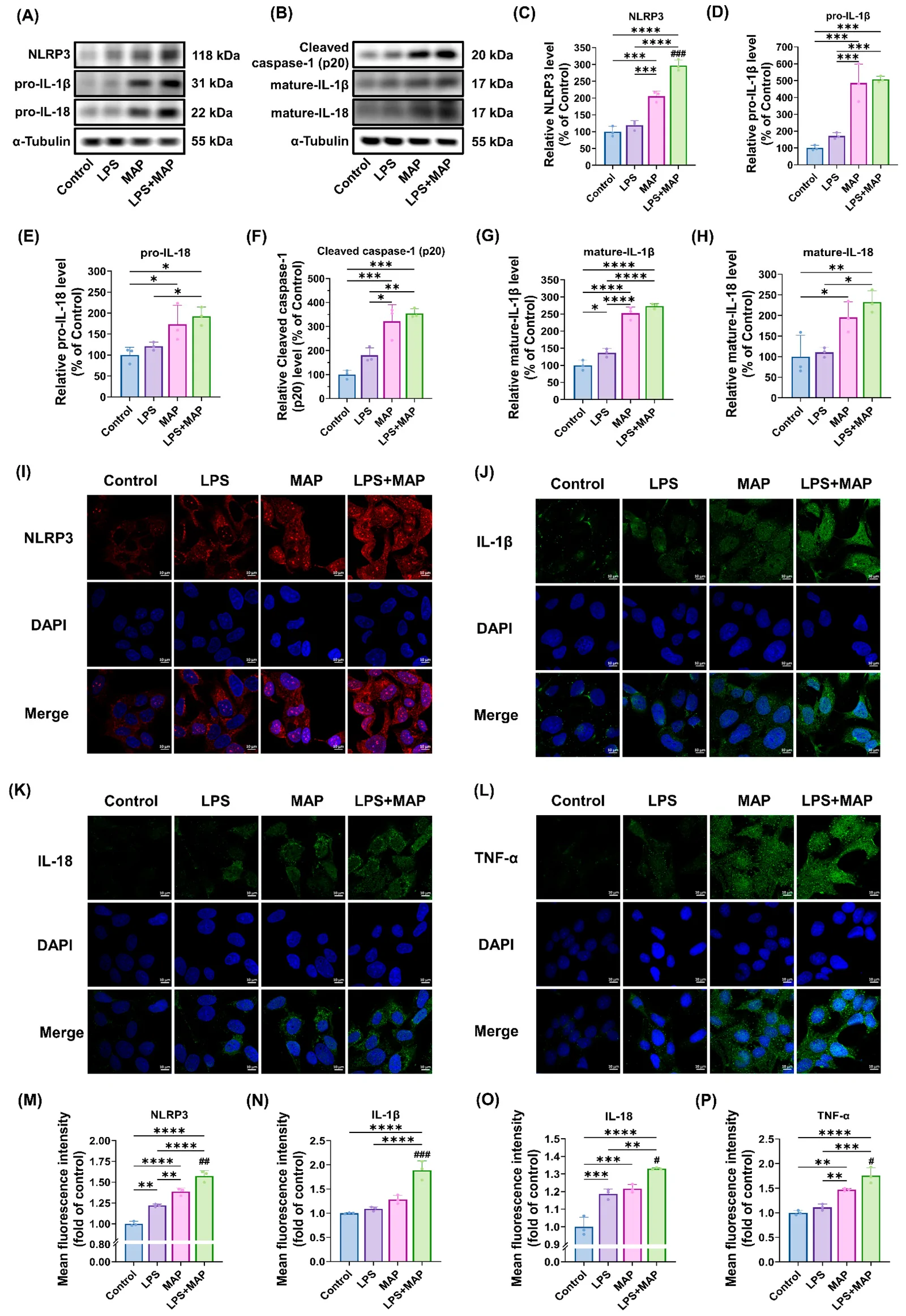
MAP crystals exacerbate cellular inflammatory response in LPS-induced inflammatory microenvironment. (**A**) Representative Western blot images of NLRP3, pro-IL-1β, and pro-IL-18. (**B**) Representative Western blot images of Cleaved caspase-1 (p20), mature-IL-1β, and mature-IL-18. (**C**–**H**) Semi-quantitative histograms of NLRP3 (**C**), pro-IL-1β (**D**), pro-IL-18 (**E**), Cleaved caspase-1 (p20) (**F**), mature-IL-1β (**G**), and mature-IL-18 (**H**), based on the Western blot results shown in (**A**,**B**). (**I**–**L**) Representative immunofluorescence images of NLRP3 (**I**), IL-1β (**J**), IL-18 (**K**), and TNF-α (**L**). Scale bar: 10 μm. (**M**–**P**) Semi-quantitative histograms of fluorescence intensity for NLRP3 (**M**), IL-1β (**N**), IL-18 (**O**), and TNF-α (**P**). Data are presented as Mean ± SD (n = 3 independent biological experiments). Two-way ANOVA was applied for the 2 × 2 factorial design, with Tukey’s post hoc pairwise comparisons performed; * *p* < 0.05, ** *p* < 0.01, *** *p* < 0.001, and **** *p* < 0.0001 between indicated groups; # *p* < 0.05, ## *p* < 0.01, and ### *p* < 0.001 versus MAP group.

As shown in Figure 5A–H, Western blot results indicated that the protein levels of NLRP3, pro-IL-1β, pro-IL-18, Cleaved caspase-1(p20), mature-IL-1β, and mature-IL-18 in each group were as follows: LPS + MAP group > MAP group > LPS group > control group; Figure 5I–P shows immunofluorescence data demonstrating that the protein levels of NLRP3, IL-1β, IL-18, and TNF-α exhibited the same trend: LPS + MAP group > MAP group > LPS group > control group. These results suggested that the LPS-induced inflammatory microenvironment further exacerbated the MAP crystal-induced upregulation of NLRP3 inflammasome-related proteins as well as the release of downstream inflammatory cytokines.

### 3.6. Effects of Inflammatory Microenvironment on MAP Crystal-Induced Cell Damage

The integrity of the epithelial tight junction depends on the interactions between the tight junction proteins claudins, the scaffold protein ZO-1, and the cytoskeleton [70]. Filamentous actin (F-actin) is the core component of cytoskeletal microfilaments. Disordered ZO-1 distribution and disrupted actin cytoskeletal architecture may impair urothelial barrier integrity [71], thereby facilitating penetration of exogenous irritants and aggravating cell damage.

**Figure 6:**
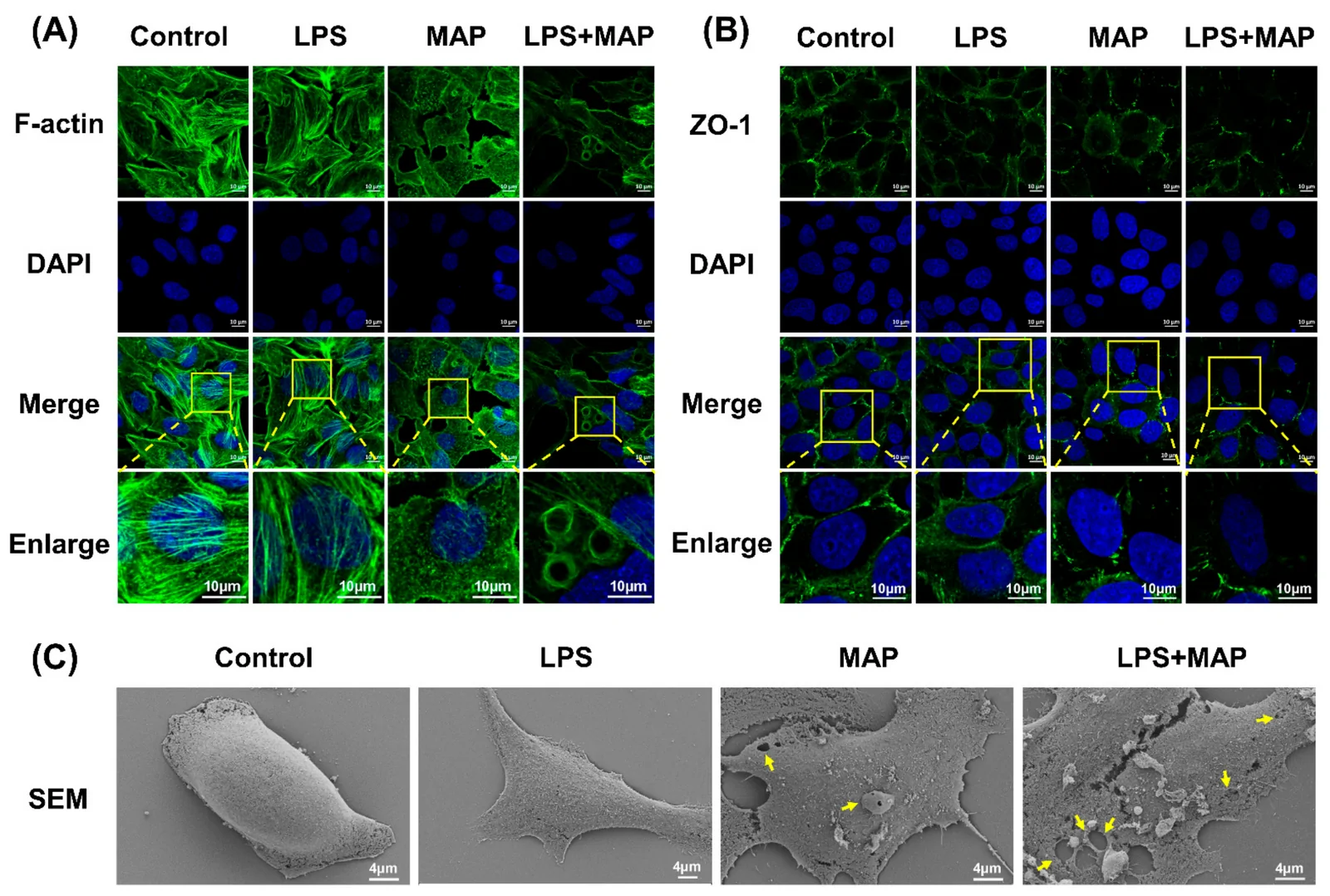
MAP crystals exacerbate cellular damage in inflammatory microenvironment. (**A**) Representative fluorescence images of intracellular microfilament morphology and distribution. Scale bar: 10 μm (n = 3 independent biological experiments). (**B**) Representative immunofluorescence images of ZO-1 expression. Scale bar: 10 μm (n = 3 independent biological experiments). (**C**) Representative SEM images of cell membrane morphology, with yellow arrows pointing to damaged cell membrane. Scale bar: 4 μm (n = 3 independent biological experiments).

As visualized in Figure 6A, cells in the control group exhibited plump morphologies with intact actin microfilament structures and distinct cellular outlines. Mild cellular shrinkage and partial F-actin depolymerization were observed in the LPS group. F-actin signals appeared blurred and disorganized in the MAP group. Severe destruction of the cytoskeletal network was detected in the LPS + MAP group, accompanied by obvious pore-like structural defects. These morphological observations indicated that the LPS-induced inflammatory microenvironment could amplify MAP crystal-mediated cytoskeletal remodeling.

As shown in Figure 6B, ZO-1 staining displayed a continuous reticular distribution along cell borders in the control group, delineating intact cellular contours and indicating well-maintained tight junction structures. The ZO-1 fluorescence signal was attenuated in the LPS group, yet relatively intact cell outlines remained observable. Discontinuous ZO-1 staining was observed in the MAP group. Notably, the ZO-1 fluorescence signal was markedly diminished in the LPS + MAP group, with discrete regions presenting a complete loss of ZO-1 signals and distinct intercellular fissures. This suggested that the LPS-induced inflammatory microenvironment exacerbated the MAP crystal-induced disruption of the continuous reticular ZO-1 structure.

As shown in the SEM image of Figure 6C, normal cells exhibited intact, plump cellular contours. Cells exhibited a spindle-shaped morphology after LPS treatment. Irregular cellular edges and partial membrane damage were observed in the MAP group, while far more severe membrane injury, highly irregular margins, and rough membrane surfaces were observed in the LPS + MAP group. These results indicated that the LPS-induced inflammatory microenvironment aggravated MAP crystal-induced abnormalities of the cell membrane surface architecture.

### 3.7. Effects of Inflammatory Microenvironment on MAP Crystal–Cell Adhesion

**Figure 7:**
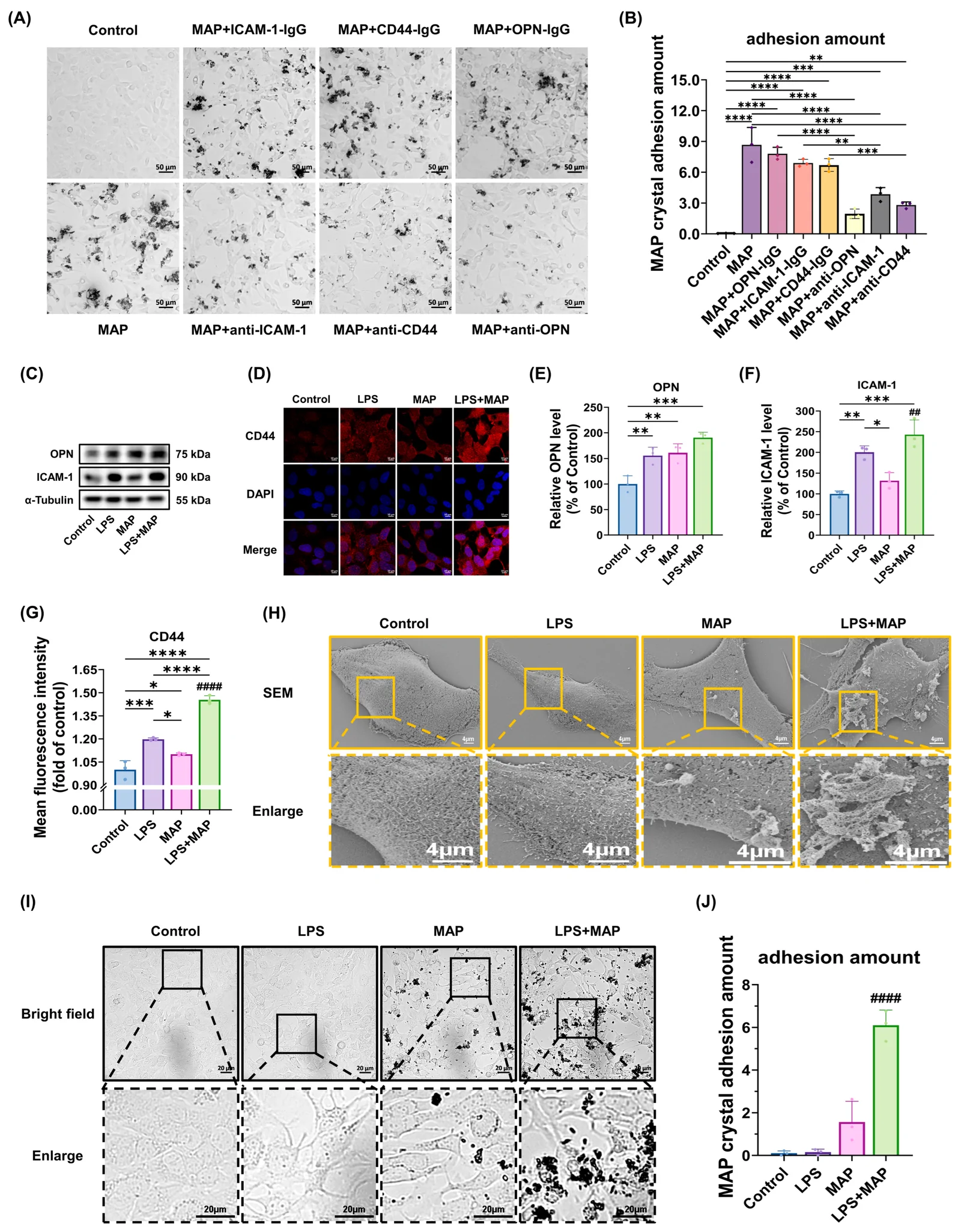
Detection of adhesion proteins and observation of MAP crystal adhesion. (**A**,**B**) Representative images of MAP crystal adhesion in neutralizing-antibody-blocking assay (**A**) and corresponding semi-quantitative histograms of crystal adhesion (**B**). Scale bar: 50 μm. (**C**) Representative Western blot images of OPN and ICAM-1. (**D**) Representative immunofluorescence images of CD44. Scale bar: 10 μm. (**E**,**F**) Semi-quantitative histograms of OPN (**E**) and ICAM-1 (**F**). (**G**) Semi-quantitative histograms of CD44. (**H**) Representative SEM images of crystal–cell adhesion. Scale bar: 4 μm. (**I**,**J**) Representative bright-field images of crystal adhesion (**I**) and corresponding semi-quantitative histograms of crystal adhesion (**J**). Scale bar: 20 μm. Data are presented as Mean ± SD (n = 3 biological independent experiments). Two-way ANOVA was applied for the 2 × 2 factorial design, with Tukey’s post hoc pairwise comparisons performed. One-way ANOVA with Tukey’s post hoc test was applied for multiple-group comparisons; * *p* < 0.05, ** *p* < 0.01, *** *p* < 0.001, and **** *p* < 0.0001 between indicated groups; ## *p* < 0.01 and #### *p* < 0.0001 versus MAP group.

Antibody-blocking intervention assays were performed to clarify the specificity and necessity of the adhesion molecules OPN, ICAM-1, and CD44 during the adhesion between MAP crystals and urothelial SV-HUC-1 cells. As presented in Figure 7A,B, pretreatment with anti-OPN, anti-ICAM-1 or anti-CD44 significantly reduced the adhesion level of MAP crystals on the cell surface compared with the corresponding IgG control group (*p* values of <0.0001, 0.0027 and 0.0002, respectively). Among them, MAP crystal adhesion levels decreased most significantly after OPN blockade. This result indicated that OPN, ICAM-1, and CD44 participated in MAP crystal–cell adhesion, and the reduction in crystal adhesion was more pronounced upon OPN blockade.

To explore the regulatory effect of the inflammatory microenvironment on the expression of these molecules, the expression levels of the adhesion molecules OPN, ICAM-1, and CD44 in each group were detected. MAP crystal adhesion on the surface was observed using scanning electron microscopy and an inverted fluorescence microscope. The experimental results showed that the LPS + MAP group exhibited the highest expression levels of adhesion molecules and had the greatest extent of MAP crystal adhesion. This finding suggested that the LPS-induced inflammatory microenvironment significantly promoted MAP crystal adhesion to SV-HUC-1 cells.

## 4. Discussion

### 4.1. Rationality Analysis of MAP Crystal Modeling Concentrations

Consistent with our previous laboratory findings [48], MAP crystals were partially dissolved in culture medium at concentrations below 800 μg/mL, accompanied by low cytotoxicity. When the concentration of MAP-3 μm crystals increased from 1000 μg/mL to 1600 μg/mL, the cell viability declined from 77.7 ± 5.1% to 35.9 ± 1.2%, presenting a prominent dose–effect relationship. Our present data align closely with these prior observations. Over MAP concentrations from 0 to 1.4 mg/mL, relative cell viability decreased in a concentration-dependent manner (Figure 2A). At 1.0 mg/mL of MAP, the cell viabilities of the non-LPS-primed and LPS-primed groups were approximately 58.81% and 56.96%, respectively (Figure 3C), which were highly similar, indicating that this concentration ensured that subsequent experiments were carried out with comparable cytotoxicity and acceptable cell viability. It should be noted that although partial crystal dissolution occurs at 1.0 mg/mL MAP during incubation, substantial amounts of MAP crystals remain. Although small amounts of ions are released into the culture medium, our previously published study has demonstrated that MAP-saturated medium induces minimal cellular damage [72]. Therefore, such trace dissolution is insufficient to trigger extensive cellular inflammation and injury.

From the perspective of clinical pathological features, MAP crystals rapidly aggregate and grow shortly after nucleation initiates [7,73]. MAP stones generally present as staghorn calculi with a high stone burden in vivo. Keller et al. [74] analyzed 18,029 spontaneously passed urinary stones and found that the average diameter (7.9 mm) and volume (61.0 mm^3^) of MAP stones were 2.2-fold and 6.8-fold larger than those of calcium oxalate monohydrate (COM) stones (3.6 mm/9.0 mm^3^), respectively. Another study [75] further illustrated that high-burden calculi were strongly associated with phosphate stones (especially struvite), while oxalate stones mostly presented with low stone burden.

### 4.2. MAP Crystals Induce Cellular Inflammation via the ROS–CTSB–NLRP3 Signaling Pathway

Current mechanistic studies on crystal-induced inflammation in the urinary tract predominantly focus on calcium oxalate (CaOx) crystals. Our findings indicated that MAP crystals share partial mechanistic similarities with CaOx crystals in triggering inflammation, and both can trigger ROS production via adhesion-mediated cellular injury and initiate inflammatory cascades [76,77,78,79]. Excess ROS impairs lysosomal membrane integrity and triggers lysosomal membrane permeabilization [80,81,82], facilitating CTSB release and NLRP3 inflammasome activation [45,83,84]. The activated NLRP3 inflammasome further cleaves and activates caspase-1, drives the maturation and secretion of pro-inflammatory cytokines, including IL-1β and IL-18 [85,86], and simultaneously upregulates the pro-inflammatory mediator TNF-α, ultimately leading to sustained and amplified inflammatory responses. Preliminary pharmacological intervention experiments were performed in this study, and the results supported that the ROS–CTSB–NLRP3 signaling axis serves as a potential pathway whereby MAP crystals induce inflammatory responses in SV-HUC-1 cells. This observation aligns with the reported mechanism underlying CaOx crystal-mediated inflammatory activation [87,88].

CaOx and MAP stones are two common types of urinary stones, differing significantly in their formation mechanisms and the inflammatory responses they induce. CaOx stones are typical metabolic calculi. When the urinary CaOx concentration reaches supersaturation, crystals precipitate and adhere to renal papillary surfaces, predominantly anchoring to Randall’s calcification plaques as nucleation sites and growth substrate, gradually depositing and growing to form stones [89]. In terms of pathological inflammatory mechanisms, CaOx crystals can acts as endogenous damage-associated molecular patterns (DAMPs) and trigger sterile inflammation independent of pathogens, which is an important mechanism by which CaOx stones cause kidney damage [41].

MAP stones are typical infectious stones, and bacterial infection is one of the key factors in their formation. Moreover, bacterial infection can activate host immune responses and synergistically amplify inflammatory damage [90]. Unlike the origin pattern of renal papillary Randall’s plaques in CaOx stones, MAP crystals nucleate and accumulate around infectious lesions in collecting ducts. As the lesions progress, crystals occupy the collecting system and eventually develop into staghorn calculi. There is no significant correlation between the progression of MAP stones and renal papillary Randall’s calcification. However, clinical studies have shown that renal interstitial inflammation occurs in 43.5% of patients with MAP stones, far higher than the 7.3% rate among patients with CaOx stones [91].

### 4.3. LPS-Induced Inflammatory Microenvironment Facilitates MAP Crystal-Induced Cell Damage

Urothelial barrier integrity relies on the coordinated crosstalk between the cytoskeleton and tight junction complexes. The actin cytoskeleton binds to the scaffold protein ZO-1 to sustain the network of tight junctions and cellular polarity, forming the structural foundation of the epithelial barrier [92]. Studies [93] have shown that inflammatory cytokines such as TNF-α and IL-1β induce cytoskeletal remodeling and suppress tight junction protein expression. Gan et al. [94] demonstrated that calcium oxalate crystals can disrupt tight junctions by remodeling F-actin, thereby resulting in the loss of epithelial cell polarity and increased paracellular permeability.

Morphological observations in this study revealed that the LPS-induced inflammatory microenvironment aggravated F-actin depolymerization and disorganization, disrupted the continuous reticular structure of ZO-1, and exacerbated cell membrane damage. Damaged cells become more vulnerable to invasion by exogenous particles such as crystals, which facilitates crystal adhesion and aggregation, an important step in kidney stone formation [95,96]. The results of this study supported that the LPS-induced inflammatory microenvironment aggravated urothelial injury while simultaneously increasing MAP crystal adhesion, which may facilitate the formation of MAP stones.

### 4.4. LPS-Induced Inflammatory Microenvironment Promotes MAP Crystal–Cell Adhesion

SV-HUC-1 cells were treated with LPSs to simulate the inflammatory microenvironment in this study. LPSs can induce cellular inflammatory responses and promote the NLRP3-dependent inflammatory signaling pathway as well as the secretion of inflammatory cytokines [97,98]. Crystal adhesion to the cell surface is a key event in the formation of kidney stones [99]. The results of this study suggested that the inflammatory microenvironment promoted the adhesion process between MAP crystals and SV-HUC-1 cells.

OPN, ICAM-1 and CD44 are classic adhesion molecules in kidney stone pathogenesis [100,101,102,103]. This study elucidated the adhesion regulatory mechanism of MAP crystals by combining protein expression analysis with antibody-blocking experiments. The results indicated that the adhesion molecules CD44, ICAM-1, and OPN participated in MAP crystal adhesion to SV-HUC-1 cells. MAP crystals mainly promoted crystal adhesion by upregulating OPN expression and relying on its function. LPS pretreatment upregulated OPN, CD44 and ICAM-1 expression levels, which may represent a potential mechanism for its enhancement of MAP crystal adhesion.

CD44 and ICAM-1 are transmembrane adhesion receptors, mainly mediating the interactions between cells and cells or between cells and the extracellular matrix [34,104]. Besides participating in intercellular interactions, OPN acts as a secreted bridging molecule, which can not only attach to CaOx crystals via its calcium-binding domain [105] but also interact with cell surface receptors such as CD44 and integrins [106], thereby facilitating crystal–cell adhesion and intracellular signal transduction.

Studies have shown that OPN contains abundant phosphorylated residues and carries dense negative charges. OPN can form electrostatic complexes with calcium ions on calcium oxalate crystal surfaces, stably adsorb to the crystal surface and inhibit crystal growth [107]. Moreover, OPN preferentially adsorbs to the (100)/(122) edges and (100) crystal planes of COM crystals with high Ca^2+^ density [108]; meanwhile, the carboxyl groups distributed along its peptide chain specifically interact with cations within the crystal Stern layer to modulate crystal adhesion and growth kinetics [109]. Notably, the (101) and (010) planes of MAP crystal exhibit high Mg^2+^ density [110]. Similar to the Ca^2+^-rich crystal planes of COM crystals, these planes also form regions enriched with local positive charges. On the basis of this shared surface charge characteristic, we hypothesize that the negatively charged adhesion molecule OPN binds to MAP crystals via electrostatic interactions. Previous studies have also suggested that stone crystals are more likely to bind to HA/OPN rather than CD44 [103]. These results combined with the results of this blocking experiment suggest that OPN may serve as a key molecule participating in the adhesion of MAP crystals to urothelial cells. However, the direct physicochemical binding mechanism between OPN and MAP crystals remains to be further verified.

### 4.5. Achievements, Limitations and Perspectives

In recent years, the role of inflammatory-related mechanisms in urinary stones has attracted growing research attention. However, current studies have either focused on isolated signaling cascades governing crystal–cell interactions [111,112,113], adopted simplified in vitro cell models with single crystal stimulation [114,115,116], or solely investigated crystal–cell adhesive behaviors [117,118]. On this basis, this study provides an integrated framework connecting NLRP3 inflammasome activation, LPS-induced epithelial injury, and enhanced crystal adhesion, which broadens the mechanistic understanding of the interactions between MAP crystals and urothelial cells in struvite stone pathogenesis.

**Figure 8:**
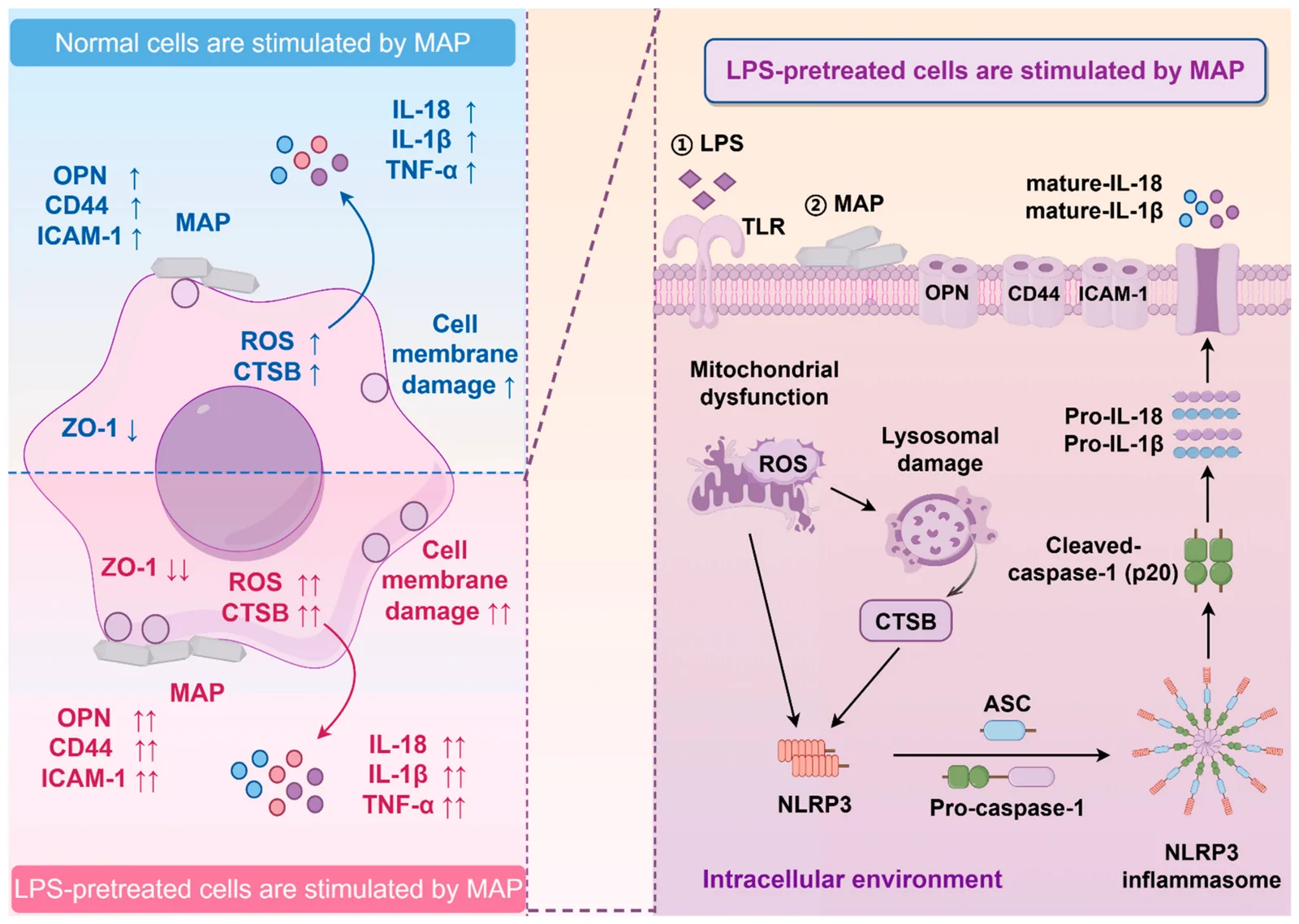
Potential-mechanism diagram illustrating how LPS pretreatment exacerbates inflammatory damage and adhesion enhancement induced by MAP crystals (by figdraw.com: www.figdraw.com).

Infectious stones are primarily composed of magnesium ammonium phosphate (MAP) and carbonate apatite (CA) [119,120]. CA crystals trigger inflammatory responses via the NLRP3 inflammasome cascade [121,122], yet it remains unclear whether this process depends on the ROS–CTSB–NLRP3 signaling axis. Distinct from CA crystals, MAP crystals possess unique pro-inflammatory properties beyond NLRP3 inflammasome activation, including the induction of extracellular trap and migrasome formation in THP-1 macrophages [73]. For the first time, the present study verifies that MAP crystals provoke urothelial inflammation in SV-HUC-1 cells via the ROS–CTSB–NLRP3 axis and preliminarily elucidates the underlying mechanism by which the LPS-induced inflammatory microenvironment exacerbates cellular injury and crystal adhesion (Figure 8). These findings provide novel mechanistic insights into MAP stone pathogenesis. Nevertheless, several limitations of this study warrant discussion.

Firstly, this study only conducted in vitro experiments using immortalized SV-HUC-1 urothelial cells. Biological characteristics such as the growth properties and genetic stability of these immortalized cell lines differ from primary urothelial cells, which limits the applicability of the research conclusions. Subsequent verification can be carried out using primary uroepithelial cell models. Primary uroepithelial cells recapitulate the authentic in vivo cellular phenotype, better reflecting the urothelial responses to MAP crystals and LPS stimulation and further improving the clinical relevance of the mechanism. Furthermore, mouse struvite stone models induced by urease-producing bacteria such as *Proteus mirabilis* can be established. In vivo intervention with NLRP3 inhibitors or the targeted regulation of OPN, CD44 and ICAM-1 will help verify our core findings through histological staining, molecular expression detection and crystal retention analysis.

Secondly, in vitro pathological simulation contains inevitable simplifications. The formation of MAP stones is closely related to infection by urease-positive bacteria. Bacteria decompose urea via urease to alkalinize urine and drive MAP crystal precipitation. In vivo stone pathogenesis involves multiple interwoven factors: bacterial biofilms, secreted metabolites, extracellular vesicles, various urinary proteins, as well as the recruitment and activation of host immune cells under continuous urinary flow [3,4,5,6]. However, this study only used a single LPS stimulus and a fixed MAP crystal concentration, which can only reflect crystal–cell interactions in the LPS-induced inflammatory microenvironment and cannot fully reproduce the dynamic and complex pathological process of stone formation. Follow-up experiments with gradient concentrations of LPS and MAP crystals, bacterial co-culture systems, and biofilm models will complement and refine the conclusions.

Thirdly, the experimental strategies adopted to validate molecular mechanisms are relatively limited. Regulatory relationships within the inflammatory signaling axis were primarily verified via pharmacological inhibitors, lacking complementary genetic validation such as gene knockdown or knockout assays. Moreover, we did not set up TLR4 blockade control groups or conduct multi-dimensional verification. It remains unclear whether LPSs induce non-specific cellular stress through TLR4-independent pathways, which requires further investigation in future studies.

Fourthly, neutralizing-antibody-blocking assays were not performed under LPS + MAP dual-stimulation conditions. Therefore, the causal relationship between the upregulated expression of OPN, CD44, and ICAM-1 and enhanced MAP crystal adhesion in the LPS-induced inflammatory microenvironment remains inferential, and this combined adhesion mechanism requires further validation in future studies.

## 5. Conclusions

This study demonstrated that the LPS-induced inflammatory microenvironment promotes MAP crystal adhesion to urothelial cells and exacerbates MAP crystal-mediated injury to SV-HUC-1 cells. MAP crystals induce inflammatory responses in SV-HUC-1 cells via the ROS–CTSB–NLRP3 signaling pathway, and the inflammatory microenvironment further strengthens the adhesion and retention of MAP crystals. These findings provide a theoretical basis for elucidating the interaction mechanism between MAP crystals and urothelium and suggest that the therapeutic targeting of inflammatory pathways and molecules related to crystal adhesion may become a promising strategy for preventing magnesium ammonium phosphate stone formation.

## Data Availability

The original contributions presented in this study are included in the article. Further inquiries can be directed to the corresponding authors.

## Supplementary Materials

The following supporting information can be downloaded at: https://www.mdpi.com/article/10.3390/cells15211983/s1, Figure S1. Uncropped Western blot images in Figure 2D; Figure S2. Uncropped Western blot images in Figure 2H; Figure S3. Uncropped Western blot images in Figure 4B; Figure S4. Uncropped Western blot images in Figure 5A; Figure S5. Uncropped Western blot images in Figure 5B; Figure S6. Uncropped Western blot images in Figure 7C.

## Abbreviations

The following abbreviations are used in this manuscript:

LPS	Lipopolysaccharide
MAP	Magnesium ammonium phosphate
CaOx	Calcium oxalate
NAC	N-acetylcysteine
SV-HUC-1	SV-40 immortalized human urothelial cell line
OPN	Osteopontin
ROS	Reactive oxygen species
CTSB	Cathepsin B
NLRP3	NOD-like receptor thermal protein domain associated protein 3
Caspase-1	Cysteine-requiring aspartate-specific protease-1
IL-1β	Interleukin-1 beta
IL-18	Interleukin-18
IL-6	Interleukin-6
TNF-α	Tumor necrosis factor-alpha
NF-κB	Nuclear factor-kappa B
AO	Acridine orange
F-actin	Filamentous actin
ZO-1	Zonula occludens-1
PBS	Phosphate-buffered saline
CA	Carbonate apatite

## References

[1] Li Z.Y., Zhang Z.C., Yu P., Ni Y.L. (2024). Microbial communities, antibiotic resistance genes, and virulence factors in urinary infectious stone-associated urinary tract infections. Urolithiasis.

[2] Al-Tulaibawi N.A.J., AL-Nussairawi M., AL-Zuhairy N.A.S. (2024). Molecular and biochemical detection of bacteria in adult patients with urinary tract infection associate renal stones. J. Ren. Inj. Prev..

[3] Das P., Gupta G., Velu V., Awasthi R., Dua K., Malipeddi H. (2017). Formation of struvite urinary stones and approaches towards the inhibition-A review. Biomed. Pharmacother..

[4] Flores C., Rohn J.L. (2025). Bacterial adhesion strategies and countermeasures in urinary tract infection. Nat. Microbiol..

[5] Flores-Mireles A.L., Walker J.N., Caparon M., Hultgren S.J. (2015). Urinary tract infections: Epidemiology, mechanisms of infection and treatment options. Nat. Rev. Microbiol..

[6] Timm M.R., Russell S.K., Hultgren S.J. (2025). Urinary tract infections: Pathogenesis, host susceptibility and emerging therapeutics. Nat. Rev. Microbiol..

[7] Espinosa-Ortiz E.J., Eisner B.H., Lange D., Gerlach R.B. (2019). Current insights into the mechanisms and management of infection stones. Nat. Rev. Urol..

[8] Torzewska A., Budzyńska A., Białczak-Kokot M., Różalski A. (2014). In vitro studies of epithelium-associated crystallization caused by uropathogens during urinary calculi development. Microb. Pathog..

[9] Mathoera R.B., Kok D.J., Verduin C.M., Nijman R.J.M. (2002). Pathological and therapeutic significance of cellular invasion by *Proteus mirabilis* in an enterocystoplasty infection stone model. Infect. Immun..

[10] Akele O., Rana F., Acharya S., LeDoux D., Chalhoub M. (2026). Targeting lipopolysaccharides in gram-negative sepsis: Therapeutic advances and challenges. J. Drug Target..

[11] Hernández-Cuellar E., Tsuchiya K., Medina-Contreras O., Valle-Ríos R. (2025). The role of inflammasomes in LPS and gram-negative bacterial sepsis. J. Clin. Med..

[12] Gerber-Tichet E., Blanchet F.P., Majzoub K., Kremer E.J. (2025). Toll-like receptor 4–a multifunctional virus recognition receptor. Trends Microbiol..

[13] Huang G.W., Bao J.W., Shao X.H., Zhou W.Y., Wu B., Ni Z.H., Wang L. (2020). Inhibiting pannexin-1 alleviates sepsis-induced acute kidney injury via decreasing NLRP3 inflammasome activation and cell apoptosis. Life Sci..

[14] An S., Yao Y., Hu H.B., Wu J.J., Li J.X., Li L.L., Wu J., Sun M.M., Deng Z.Y., Zhang Y.Y. (2023). PDHA1 hyperacetylation-mediated lactate overproduction promotes sepsis-induced acute kidney injury via Fis1 lactylation. Cell Death Dis..

[15] Chen D., Xiong X.Q., Zang Y.H., Tong Y., Zhou B., Chen Q., Li Y.H., Gao X.Y., Kang Y.M., Zhu G.Q. (2017). BCL6 attenuates renal inflammation via negative regulation of NLRP3 transcription. Cell Death Dis..

[16] McAleer I.M., Kaplan G.W., Bradley J.S., Carroll S.F., Griffith D.P. (2003). Endotoxin content in renal calculi. J. Urol..

[17] Wang W.B., Chen I.C., Jiang S.S., Chen H.R., Hsu C.Y., Hsueh P.R., Hsu W.B., Liaw S.J. (2008). Role of RppA in the regulation of polymyxin B susceptibility, swarming, and virulence factor expression in *Proteus mirabilis*. Infect. Immun..

[18] Wang C.R., Ge F.H., Ge F.J., Xu Z.H., Jiang J.L. (2025). Harnessing stem cell therapeutics in LPS-induced animal models: Mechanisms, efficacies, and future directions. Stem. Cell Res. Ther..

[19] Zhang X., Tian X., Wang Y., Yan Y., Wang Y., Su M.C., Lv H.F., Li K.T., Hao X.B., Xing X. (2024). Application of lipopolysaccharide in establishing inflammatory models. Int. J. Biol. Macromol..

[20] Calmasini F.B., Alexandre E.C., Oliveira M.G., Silva F.H., Soares A.G., Costa S.K.P., Antunes E. (2021). Lipopolysaccharide reduces urethral smooth muscle contractility via cyclooxygenase activation. J. Physiol. Biochem..

[21] Chen M., Tang Y.B., Fu Y., Hu H.W., Cui E.D., Wen Z.L., Zhong W., Su J.M., Ge B. (2024). The protective role of PGC-1α in cystitis glandularis: Mitigating mitochondrial injury and inflammation. Int. J. Clin. Pract..

[22] Ke H.W., Zhu L., Wang Q., Xu K.X. (2025). Exploring the therapeutic potential of PACAP in Hunner-type Interstitial Cystitis. World J. Urol..

[23] Verkoelen C.F., van der Boom B.G., Schröder F.H., Romijn J.C. (1997). Cell cultures and nephrolithiasis. World J. Urol..

[24] Petrović A., Kizivat T., Bilić Ćurčić I., Smolić R., Smolić M. (2021). In vitro cell culture models of hyperoxaluric states: Calcium oxalate and renal epithelial cell interactions. Crystals.

[25] Taguchi K., Okada A., Unno R., Hamamoto S., Yasui T. (2021). Macrophage function in calcium oxalate kidney stone formation: A systematic review of literature. Front. Immunol..

[26] Schilling J.D., Martin S.M., Hunstad D.A., Patel K.P., Mulvey M.A., Justice S.S., Lorenz R.G., Hultgren S.J. (2003). CD14-and Toll-like receptor-dependent activation of bladder epithelial cells by lipopolysaccharide and type 1 piliated *Escherichia coli*. Infect. Immun..

[27] Cao Y., Chen X.H., Zhu Z.J., Luo Z.L., Hao Y.Q., Yang X.Y., Feng J., Zhang Z.W., Hu J.J., Jian Y.H. (2024). STING contributes to lipopolysaccharide-induced tubular cell inflammation and pyroptosis by activating endoplasmic reticulum stress in acute kidney injury. Cell Death Dis..

[28] Li B.J., Xia Y.Q., Mei S.Q., Ye Z.H., Song B.F., Yan X.Z., Lin F.Y., Rao T., Yu W.M., Mei C. (2023). Histone H3K27 methyltransferase EZH2 regulates apoptotic and inflammatory responses in sepsis-induced AKI. Theranostics.

[29] Wang X., Yin H., Fan L., Zhou Y.Q., Tang X.L., Fei X.J., Tang H.L., Peng J., Zhang J.J., Xue Y. (2021). Shionone alleviates NLRP3 inflammasome mediated pyroptosis in interstitial cystitis injury. Int. Immunopharmacol..

[30] Liao G.L., Long C.L., Guo R., Wang S.S., Han R.J., Yang J.H., Liu J. (2025). Conditional immortalization of mesenchymal stem cells and their extracellular vesicles therapy for interstitial cystitis/bladder pain syndrome. Stem Cell Res. Ther..

[31] Wang X., Fan L., Yin H., Zhou Y.Q., Tang X.L., Fei X.J., Tang H.L., Peng J., Ren X.Q., Xue Y. (2020). Protective effect of Aster tataricus extract on NLRP3-mediated pyroptosis of bladder urothelial cells. J. Cell. Mol. Med..

[32] Zhang J., Wang J.P., Wang X.H., Yan Z.H., Meng L.F., Zhang Y.G. (2025). Pterostilbene reduces cyclophosphamide-induced interstitial cystitis by facilitating Nrf2 activation and suppressing the NLRP3 inflammasome pathway. Int. J. Mol. Sci..

[33] Zhang Z.M., Chen C., Zhou J., Li C.H., Du X.F., Hou H., Cao M., Yu D.L., Zhang J.J., Gu J. (2025). Carboxymethyl Poria cocos polysaccharides protect against septic kidney injury by regulating the Nrf2-NF-κB signaling pathway. Int. J. Biol. Macromol..

[34] Lu C.H., Lin C.H., Li K.J., Shen C.Y., Wu C.H., Kuo Y.M., Lin T.S., Yu C.L., Hsieh S.C. (2017). Intermediate molecular mass hyaluronan and CD44 receptor interactions enhance neutrophil phagocytosis and IL-8 production via p38-and ERK1/2-MAPK signalling pathways. Inflammation.

[35] Park J.W., Jung K.H., Lee J.H., Moon S.H., Cho Y.S., Lee K.H. (2021). ^89^Zr anti-CD44 immuno-PET monitors CD44 expression on splenic myeloid cells and HT29 colon cancer cells. Sci. Rep..

[36] Zhang J., Yang G.M., Zhu Y., Peng X.Y., Li T., Liu L.M. (2018). Relationship of Cx43 regulation of vascular permeability to osteopontin-tight junction protein pathway after sepsis in rats. Am. J. Physiol. Regul. Integr. Comp. Physiol..

[37] Lee H.J., Jeong S.J., Lee H.J., Lee E.O., Bae H., Lieske J.C., Kim S.H. (2011). 1,2,3,4,6-Penta-O-galloyl-beta-D-glucose reduces renal crystallization and oxidative stress in a hyperoxaluric rat model. Kidney Int..

[38] Kanlaya R., Naruepantawart O., Thongboonkerd V. (2019). Flagellum is responsible for promoting effects of viable *Escherichia coli* on calcium oxalate crystallization, crystal growth, and crystal aggregation. Front. Microbiol..

[39] Prywer J., Torzewska A. (2019). Aggregation of poorly crystalline and amorphous components of infectious urinary stones is mediated by bacterial lipopolysaccharide. Sci. Rep..

[40] Prywer J., Torzewska A. (2019). Impact of bacteria on aggregation of crystalline and amorphous components of infectious urinary stones. J. Cryst. Growth.

[41] Swanson K.V., Deng M., Ting J.P.Y. (2019). The NLRP3 inflammasome: Molecular activation and regulation to therapeutics. Nat. Rev. Immunol..

[42] Paik S., Kim J.K., Shin H.J., Park E.J., Kim I.S., Jo E.K. (2025). Updated insights into the molecular networks for NLRP3 inflammasome activation. Cell. Mol. Immunol..

[43] Pan Y.G., Huang M.T., Sekar P., Huang D.Y., Lin W.W., Hsieh S.L. (2021). Decoy receptor 3 inhibits monosodium urate-induced NLRP3 inflammasome activation via reduction of reactive oxygen species production and lysosomal rupture. Front. Immunol..

[44] Yalcinkaya M., Tall A.R. (2025). Cholesterol crystals as triggers of NLRP3 inflammasome activation in atherosclerosis. Curr. Atheroscler. Rep..

[45] Ye T., Wang C.T., Yan J.F., Qin Z.X., Qin W.P., Ma Y.X., Wan Q.Q., Lu W.C., Zhang M., Tay F.R. (2023). Lysosomal destabilization: A missing link between pathological calcification and osteoarthritis. Bioact. Mater..

[46] Liu Z.M., Luo X.G., Zhang Z.C., Zhang Q., Wang C., Chen H.S., Long C.L., Liu X., Wei G.H. (2025). MAFB-mediated CEBPA regulated human urothelium growth through Wnt/β-catenin signaling pathway. Genes Dis..

[47] Luo H.J., Zhou H., Chen Y.Z., Sun X.W., Li Y.H., Li G.J., Long S.Y., Wang S.Y., Liang G.B., Chen S.L. (2024). Hypoxia impairs urothelial barrier function by inhibiting the expression of tight junction proteins in SV-HUC-1 cells. J. Cell. Mol. Med..

[48] Tan R.M., Tong X.Y., Dai W.X., Zhang J.Y., Ouyang J.M. (2025). Synthesis, characterization and size-dependent cytotoxicity of magnesium ammonium phosphate hexahydrate crystals of different sizes on renal epithelial cells. BBA Mol. Cell Res..

[49] Khaket T.P., Singh M.P., Khan I., Kang S.C. (2020). In vitro and in vivo studies on potentiation of curcumin-induced lysosomal-dependent apoptosis upon silencing of cathepsin C in colorectal cancer cells. Pharmacol. Res..

[50] Wu J.J., Guo Q., Li J.T., Yuan H., Xiao C.T., Qiu J.G., Wu Q., Wang D.J. (2023). Loperamide induces protective autophagy and apoptosis through the ROS/JNK signaling pathway in bladder cancer. Biochem. Pharmacol..

[51] He X.L., Yang W.J., Zeng Z.J., Wei Y., Gao J., Zhang B., Li L., Liu L.Q., Wan Y., Zeng Q. (2020). NLRP3-dependent pyroptosis is required for HIV-1 gp120-induced neuropathology. Cell. Mol. Immunol..

[52] Coll R.C., Robertson A.A., Chae J.J., Higgins S.C., Muñoz-Planillo R., Inserra M.C., Vetter I., Dungan L.S., Monks B.G., Stutz A. (2015). A small-molecule inhibitor of the NLRP3 inflammasome for the treatment of inflammatory diseases. Nat. Med..

[53] Chen J., Li Q., Hong Y.F., Zhou X.Z., Yu C.J., Tian X.M., Zhao J., Long C.L., Shen L.J., Wu S.D. (2023). Inhibition of the NF-κB signaling pathway alleviates pyroptosis in bladder epithelial cells and neurogenic bladder fibrosis. Int. J. Mol. Sci..

[54] Nie H., Liu G., Lu W.H., Yan C., Huang Z.S., Zhou W.M., Li T.S. (2026). Intracellular osteopontin of macrophages promotes carotid plaques formation by inducing foam cells and releasing proinflammatory cytokines. J. Am. Heart Assoc..

[55] Liu S.L., Cheng C.H. (2017). Akt signaling is sustained by a CD44 splice isoform-mediated positive feedback loop. Cancer Res..

[56] Bharadwaj A.S., Stempel A.J., Olivas A., Franzese S.E., Ashander L.M., Ma Y., Lie S., Appukuttan B., Smith J.R. (2017). Molecular signals involved in human B cell migration into the retina: In vitro investigation of ICAM-1, VCAM-1, and CXCL13. Ocul. Immunol. Inflamm..

[57] Shihan M.H., Novo S.G., Le Marchand S.J., Wang Y., Duncan M.K. (2021). A simple method for quantitating confocal fluorescent images. Biochem. Biophys. Rep..

[58] Schneider C.A., Rasband W.S., Eliceiri K.W. (2012). NIH Image to ImageJ: 25 years of image analysis. Nat. Methods.

[59] Du H.P., Guo Y., Zhu Y.M., Gao D.F., Lin B., Liu Y., Xu Y., Said A., Khan T., Liu L.J. (2023). RIPK1 inhibition contributes to lysosomal membrane stabilization in ischemic astrocytes via a lysosomal Hsp70.1B-dependent mechanism. Acta Pharmacol. Sin..

[60] Xiong P., Zheng Y.Y., Ouyang J.M. (2023). Carboxylated Pocoa polysaccharides inhibited oxidative damage and inflammation of HK-2 cells induced by calcium oxalate nanoparticles. Biomed. Pharmacother..

[61] Han J., Tong X.Y., Zheng Y.Y., Cheng J.H., Ouyang J.M., Li K. (2025). Corn silk polysaccharides before and after selenization reduced calcium oxalate crystal-induced HK-2 cells pyroptosis by inhibiting the NLRP3-GSDMD signaling pathway. J. Inflamm. Res..

[62] Jang D.I., Lee A.H., Shin H.Y., Song H.R., Park J.H., Kang T.B., Lee S.R., Yang S.H. (2021). The role of tumor necrosis factor alpha (TNF-α) in autoimmune disease and current TNF-α inhibitors in therapeutics. Int. J. Mol. Sci..

[63] Fajgenbaum D.C., June C.H. (2020). Cytokine storm. N. Engl. J. Med..

[64] Mihara M., Hashizume M., Yoshida H., Suzuki M., Shiina M. (2012). IL-6/IL-6 receptor system and its role in physiological and pathological conditions. Clin. Sci..

[65] Ouyang L., Tang H.L., Liu Z.L., Tian Y., Gao X., Peng T., Wang Z., Lan X.Y., Shen W.J., Xiao D.F. (2023). Resveratrol inhibits LPS-induced apoptosis in bovine mammary epithelial cells: The role of PGC1α-SIRT3 axis. In Vitro Cell. Dev. Biol. Anim..

[66] Carnazzo V., Rigante D., Restante G., Basile V., Pocino K., Basile U. (2025). The entrenchment of NLRP3 inflammasomes in autoimmune disease-related inflammation. Autoimmun. Rev..

[67] Chen Y., Ye X.Y., Escames G., Lei W.R., Zhang X., Li M., Jing T., Yao Y., Qiu Z.Y., Wang Z. (2023). The NLRP3 inflammasome: Contributions to inflammation-related diseases. Cell. Mol. Biol. Lett..

[68] Hirano T. (2021). IL-6 in inflammation, autoimmunity and cancer. Int. Immunol..

[69] Kany S., Vollrath J.T., Relja B. (2019). Cytokines in inflammatory disease. Int. J. Mol. Sci..

[70] Van Itallie C.M., Tietgens A.J., Anderson J.M. (2017). Visualizing the dynamic coupling of claudin strands to the actin cytoskeleton through ZO-1. Mol. Biol. Cell.

[71] Citalan-Madrid A.F., Vargas-Robles H., Garcia-Ponce A., Shibayama M., Betanzos A., Nava P., Salins-Lara C., Rottner K., Mennigen R., Schnor M. (2017). Cortactin deficiency causes increased RhoA/ROCK1-dependent actomyosin contractility, intestinal epithelial barrier dysfunction, and disproportionately severe DSS-induced colitis. Mucosal Immunol..

[72] Tong X.Y., Nong W.J., Zhang J.Y., Ouyang J.M. (2026). Magnesium ammonium phosphate hexahydrate (struvite) crystals induce macrophage extracellular traps, migrasomes and pyroptosis. Chem. Biol. Interact..

[73] Ye Z.L., Shen Y., Ye X., Zhang Z.J., Chen S.H., Shi J.W. (2014). Phosphorus recovery from wastewater by struvite crystallization: Property of aggregates. J. Environ. Sci..

[74] Keller E.X., De Coninck V., Audouin M., Doizi S., Daudon M., Traxer O. (2019). Stone composition independently predicts stone size in 18,029 spontaneously passed stones. World J. Urol..

[75] Shapur N.K., Uvarov V., Popov I., Katz R., Gofrit O.N., Landau E.H., Pode D., Duvdevani M. (2012). Crystallite size-is it a new predictor for renal stone burden?. Urology.

[76] Sun Y., Lu Y.F., Saredy J., Wang X.W., Drummer Iv C., Shao Y., Saaoud F., Xu K.M., Liu M., Yang W.Y. (2020). ROS systems are a new integrated network for sensing homeostasis and alarming stresses in organelle metabolic processes. Redox Biol..

[77] Wang P.F., Gong Q.J., Hu J.B., Li X., Zhang X.J. (2021). Reactive oxygen species (ROS)-responsive prodrugs, probes, and theranostic prodrugs: Applications in the ROS-related diseases. J. Med. Chem..

[78] Liu H.R., Jiao Y.Q., Wang P.C., Chen Y.J., Xu M.K., Zhang X.J., Zheng X.C., Yang Z.S. (2026). Oxidative stress and antioxidant therapeutic mechanisms. Pharmacol. Ther..

[79] Liu J.T., Han X.Y., Zhang T.Y., Tian K.Y., Li Z.P., Luo F. (2023). Reactive oxygen species (ROS) scavenging biomaterials for anti-inflammatory diseases: From mechanism to therapy. J. Hematol. Oncol..

[80] Chen Y., Yang Z.Q., Wang S.B., Ma Q., Li L.Y., Wu X.J., Guo Q.Q., Tao L., Shen X.C. (2023). Boosting ROS-mediated lysosomal membrane permeabilization for cancer ferroptosis therapy. Adv. Healthc. Mater..

[81] Johansson A.C., Appelqvist H., Nilsson C., Kågedal K., Roberg K., Ollinger K. (2010). Regulation of apoptosis-associated lysosomal membrane permeabilization. Apoptosis.

[82] Zhang R., Vooijs M.A., Keulers T.G.H. (2025). Key mechanisms in lysosome stability, degradation and repair. Mol. Cell. Biol..

[83] Xie Z., Zhao M.Y., Yan C.X., Kong W., Lan F., Narengaowa, Zhao S.X., Yang Q.H., Bai Z.T., Qing H. (2023). Cathepsin B in programmed cell death machinery: Mechanisms of execution and regulatory pathways. Cell Death Dis..

[84] Zhou H.Q., Wang L., Yang Y.Y., Ye F.Z., Zhao X.Y., Zhu K.S., Huang Z.H., Lv W., Yu H.M. (2026). Cathepsin B overexpression and lysosomal leakage in inflammatory microglia promote neuroinflammation in olfactory dysfunction by triggering mitochondrial dysfunction and pyroptosis. Brain Behav. Immun..

[85] Dubey S.R., Turnbull C., Pandey A., Zhao A.Y., Kurera M., Al-Zidan R., Shen C., Gautam M., Mahajan S., Jadhav P.S. (2025). Molecular mechanisms and regulation of inflammasome activation and signaling: Sensing of pathogens and damage molecular patterns. Cell. Mol. Immunol..

[86] Vande Walle L., Lamkanfi M. (2024). Drugging the NLRP3 inflammasome: From signalling mechanisms to therapeutic targets. Nat. Rev. Drug Discov..

[87] Bai J.H., Lu T., Zhao Z., Wei F.C., He K.K., He X.L., Jing Y.M., Wang F.L., Qin W.J., Xu Z.C. (2025). The role of inflammation and ROS in CaOx kidney stones. Eur. J. Med. Res..

[88] Liu Y.L., Sun Y., Kang J.N., He Z.Q., Liu Q., Wu J.H., Li D.R., Wang X., Tao Z.W., Guan X.F. (2022). Role of ROS-induced NLRP3 inflammasome activation in the formation of calcium oxalate nephrolithiasis. Front. Immunol..

[89] Khan S.R., Canales B.K., Dominguez-Gutierrez P.R. (2021). Randall’s plaque and calcium oxalate stone formation: Role for immunity and inflammation. Nat. Rev. Nephrol..

[90] Reyes L., Alvarez S., Allam A., Reinhard M., Brown M.B. (2009). Complicated urinary tract infection is associated with uroepithelial expression of proinflammatory protein S100A8. Infect. Immun..

[91] Jaeger C.D., Rule A.D., Mehta R.A., Vaughan L.E., Vrtiska T.J., Holmes D.R., McCollough C.M., Ziegelmann M.J., Herrera Hernandez L.P., Lieske J.C. (2016). Endoscopic and pathologic characterization of papillary architecture in struvite stone formers. Urology.

[92] Svitkina T. (2018). The actin cytoskeleton and actin-based motility. Cold Spring Harb. Perspect. Biol..

[93] Haque M., Kaminsky L., Abdulqadir R., Engers J., Kovtunov E., Rawat M., Al-Sadi R., Ma T.Y. (2024). *Lactobacillus acidophilus* inhibits the TNF-α-induced increase in intestinal epithelial tight junction permeability via a TLR-2 and PI3K-dependent inhibition of NF-κB activation. Front. Immunol..

[94] Gan X.G., Xu H.T., Wang Z.H. (2022). Phosphatidylserine eversion regulated by phospholipid scramblase activated by TGF-β1/Smad signaling in the early stage of kidney stone formation. Urolithiasis.

[95] Peerapen P., Thongboonkerd V. (2021). Calcium oxalate monohydrate crystal disrupts tight junction via F-actin reorganization. Chem. Biol. Interact..

[96] Peerapen P., Thongboonkerd V. (2011). Effects of calcium oxalate monohydrate crystals on expression and function of tight junction of renal tubular epithelial cells. Lab. Investig..

[97] Chhunchha B., Kubo E., Lehri D., Singh D.P. (2025). NLRP3 inflammasome and inflammatory response in aging disorders: The entanglement of redox modulation in different outcomes. Cells.

[98] Paik S., Kim J.K., Silwal P., Sasakawa C., Jo E.K. (2021). An update on the regulatory mechanisms of NLRP3 inflammasome activation. Cell. Mol. Immunol..

[99] Alelign T., Petros B. (2018). Kidney stone disease: An update on current concepts. Adv. Urol..

[100] Huang H.S., Chen J., Chen C.F. (2000). Circulating adhesion molecules and neutral endopeptidase enzymuria in patients with urolithiasis and hydronephrosis. Urology.

[101] Wang B.H., He G.F., Xu G., Wen J.M., Yu X. (2020). miRNA-34a inhibits cell adhesion by targeting CD44 in human renal epithelial cells: Implications for renal stone disease. Urolithiasis.

[102] Verhulst A., Asselman M., Persy V.P., Schepers M.S.J., Helbert M.F., Verkoelen C.F., De Broe M.E. (2003). Crystal retention capacity of cells in the human nephron: Involvement of CD44 and its ligands hyaluronic acid and osteopontin in the transition of a crystal binding-into a nonadherent epithelium. J. Am. Soc. Nephrol..

[103] Asselman M., Verhulst A., De Broe M.E., Verkoelen C.F. (2003). Calcium oxalate crystal adherence to hyaluronan-, osteopontin-, and CD44-expressing injured/regenerating tubular epithelial cells in rat kidneys. J. Am. Soc. Nephrol..

[104] Singh V., Kaur R., Kumari P., Pasricha C., Singh R. (2023). ICAM-1 and VCAM-1: Gatekeepers in various inflammatory and cardiovascular disorders. Clin. Chim. Acta..

[105] Tanaka Y., Maruyama M., Okada A., Furukawa Y., Momma K., Sugiura Y., Tajiri R., Sawada K.P., Tanaka S., Takano K. (2021). Multicolor imaging of calcium-binding proteins in human kidney stones for elucidating the effects of proteins on crystal growth. Sci. Rep..

[106] Xie Y., Sakatsume M., Nishi S., Narita I., Arakawa M., Gejyo F. (2001). Expression, roles, receptors, and regulation of osteopontin in the kidney. Kidney Int..

[107] Grohe B., O’Young J., Ionescu D.A., Lajoie G., Rogers K.A., Karttunen M., Goldberg H.A., Hunter G.K. (2007). Control of calcium oxalate crystal growth by face-specific adsorption of an osteopontin phosphopeptide. J. Am. Chem. Soc..

[108] Kazemi-Zanjani N., Chen H.H., Goldberg H.A., Hunter G.K., Grohe B., Lagugné-Labarthet F. (2012). Label-free mapping of osteopontin adsorption to calcium oxalate monohydrate crystals by tip-enhanced Raman spectroscopy. J. Am. Chem. Soc..

[109] Grohe B., Taller A., Vincent P.L., Tieu L.D., Rogers K.A., Heiss A., Sørensen E.S., Mittler S., Goldberg H.A., Hunter G.K. (2009). Crystallization of calcium oxalates is controlled by molecular hydrophilicity and specific polyanion-crystal interactions. Langmuir.

[110] Li H., Yao Q.Z., Wang Y.Y., Li Y.L., Zhou G.T. (2015). Biomimetic synthesis of struvite with biogenic morphology and implication for pathological biomineralization. Sci. Rep..

[111] Gan L.F., Li W., Luo P.Y., Zhang F.T., Zhong H.D., Xie M.G., Lu Y.R., Qian B., Zheng L.Y. (2026). CaSR regulates SLC26A6 expression via the PKA-FOXO4 signaling axis to promote experimental calcium oxalate kidney stone formation in rats. Front. Cell Dev. Biol..

[112] Yan X.Z., Xia Y.Q., Li B.J., Ye Z.H., Li L., Yuan T.H., Song B.F., Yu W.M., Rao T., Ning J.Z. (2024). The SOX4/EZH2/SLC7A11 signaling axis mediates ferroptosis in calcium oxalate crystal deposition-induced kidney injury. J. Transl. Med..

[113] Ju Y.J., Du M.W., Wang Z.Y., Mu X., Miao Y.D., Guo Z.M., Wang D.K., Wang S.Y., Ding J.J., Jin G.W. (2024). Kukoamine A alleviates nephrolithiasis by inhibiting endogenous oxalate synthesis via the IL-6/JAK/STAT3/DAO signaling pathway. Phytomedicine.

[114] Peerapen P., Putpeerawit P., Boonmark W., Thongboonkerd V. (2024). Resveratrol inhibits calcium oxalate crystal growth, reduces adhesion to renal cells and induces crystal internalization into the cells, but promotes crystal aggregation. Curr. Res. Food Sci..

[115] Yuan T.H., Ye Z.H., Mei S.Q., Zhang M., Wu M., Lin F.Y., Yu W.M., Li W., Zhou X.J., Cheng F. (2025). PRMT1-mediated methylation of UBE2m promoting calcium oxalate crystal-induced kidney injury by inhibiting fatty acid metabolism. Cell Death Dis..

[116] Li S., Zhou D.H., Zhu Z.X., Tan X.Y., Tang W.W., Gong J.B. (2023). Boosting inhibition performance of natural polyphenols for the prevention of calcium oxalate kidney stones through synergistic cooperativity. Commun. Mater..

[117] Peerapen P., Rattananinsruang P., Putpeerawit P., Boonmark W., Thongboonkerd V. (2025). The direct inhibitory effects of an antioxidant, N-acetylcysteine, against calcium oxalate crystal growth, aggregation and adhesion to MDCK renal cells. Food Chem. Toxicol..

[118] Hadpech S., Srinarawat W., Thongboonkerd V. (2025). Promoting activities of human cyclophilin A on calcium oxalate stone formation at crystal growth, aggregation and crystal-cell adhesion phases. Int. J. Biol. Macromol..

[119] Thomas B., Tolley D. (2008). Concurrent urinary tract infection and stone disease: Pathogenesis, diagnosis and management. Nat. Clin. Pract. Urol..

[120] Bargagli M., Scoglio M., Howles S.A., Fuster D.G. (2025). Kidney stone disease: Risk factors, pathophysiology and management. Nat. Rev. Nephrol..

[121] Herman J., Le Goff B., De Lima J., Brion R., Chevalier C., Blanchard F., Darrieutort-Laffite C. (2021). Pro-inflammatory effects of human apatite crystals extracted from patients suffering from calcific tendinopathy. Arthritis Res. Ther..

[122] Klück V., Boahen C.K., Kischkel B., Dos Santos J.C., Matzaraki V., Boer C.G., van Meurs J.B.J., Schraa K., Lemmers H., Dijkstra H. (2023). A functional genomics approach reveals suggestive quantitative trait loci associated with combined TLR4 and BCP crystal-induced inflammation and osteoarthritis. Osteoarthr. Cartil..

