# Supplementary material for: LPS-Induced Inflammatory Microenvironment Aggravates Urothelial Cell Damage and MAP Crystal Adhesion
Source: Cells. 2026 Oct 30;15(21):1983. doi: 10.3390/cells15211983 (PMC13762146; doi:10.3390/cells15211983)
Supplement: Supplementary file 1 [file cells-15-01983-s001.zip › cells-4558914-supplementary.pdf]

Supplementary Materials

Figure 2D

(D)

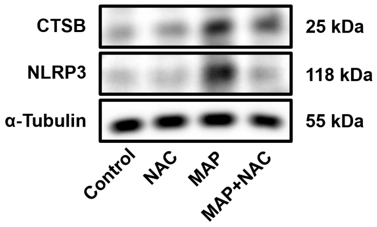

1-CTSB

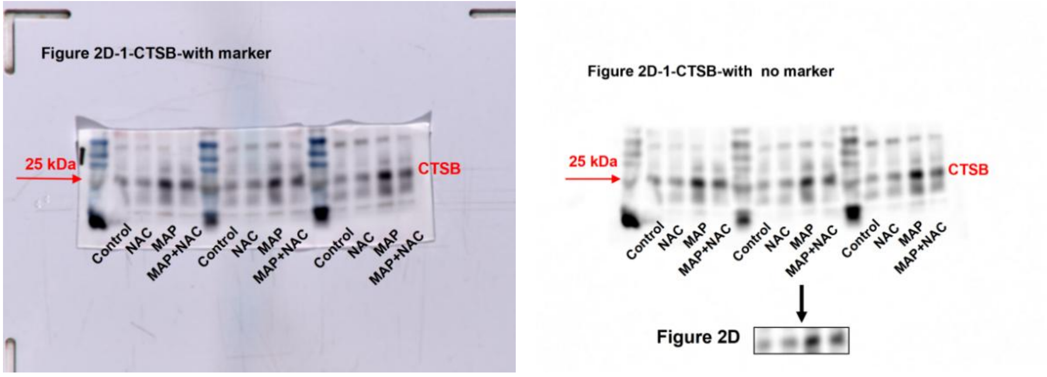

2-NLRP3

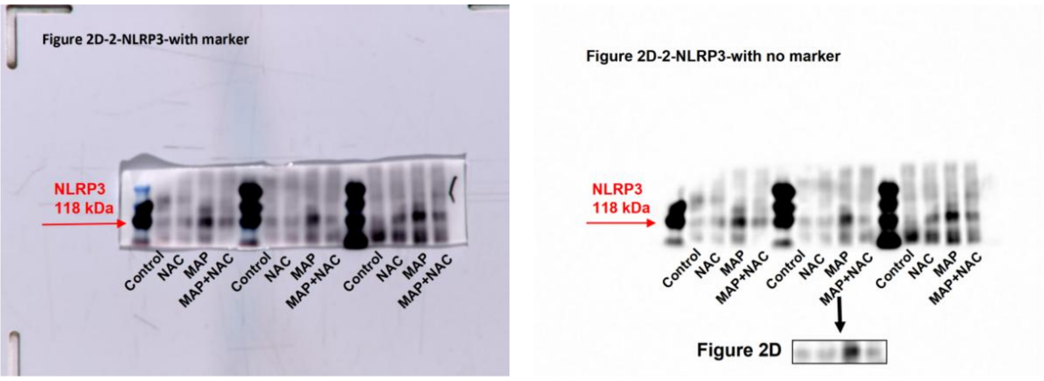

3-α-Tubulin

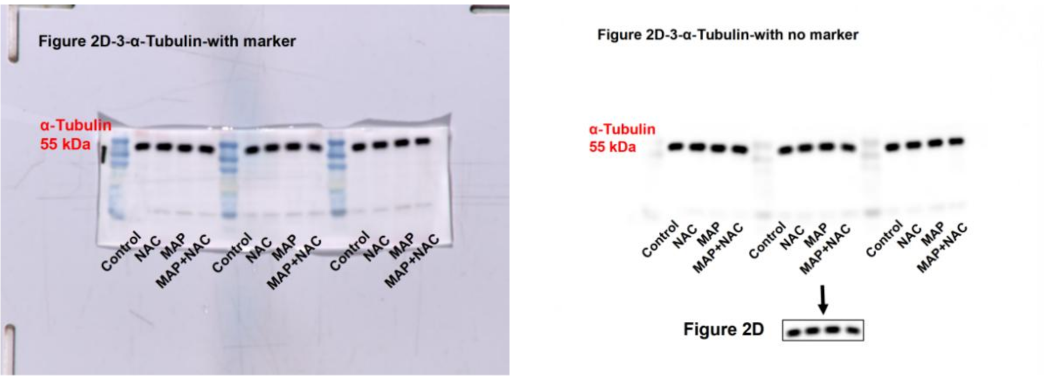

Figure S1. Uncropped Western blot images in Figure 2D.

Figure 2H

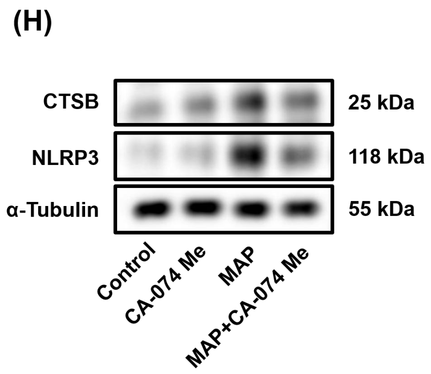

1-CTSB

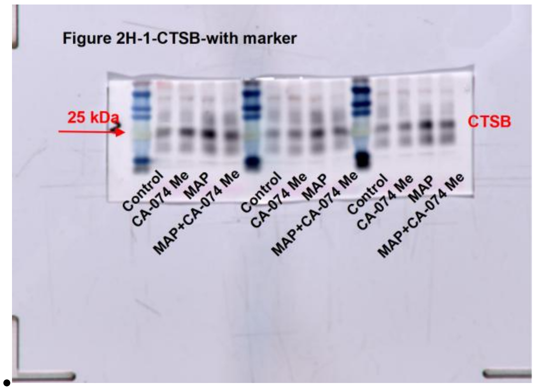

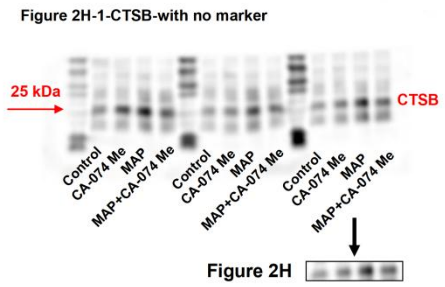

2-NLRP3

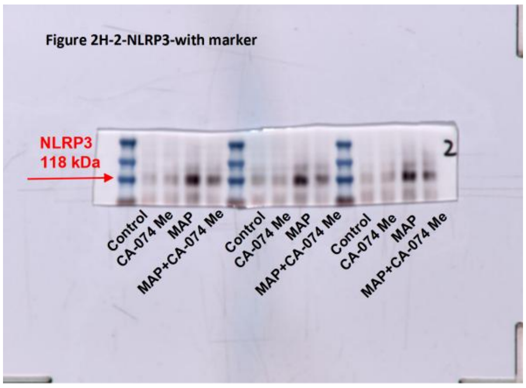

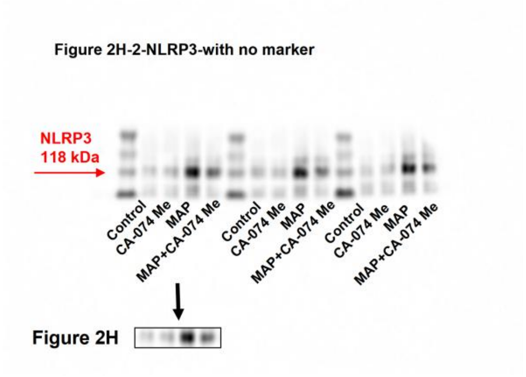

3-α-Tubulin

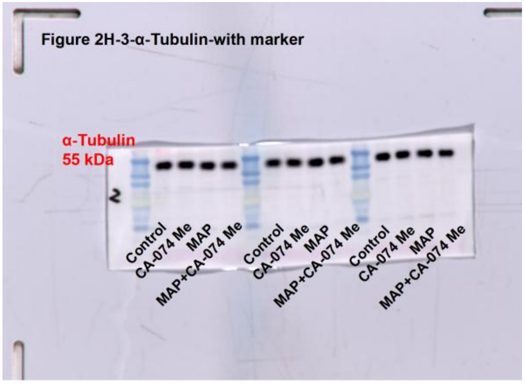

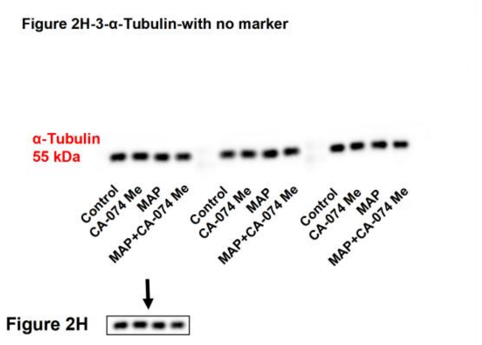

Figure S2. Uncropped Western blot images in Figure 2H.

Figure 4B

(B)

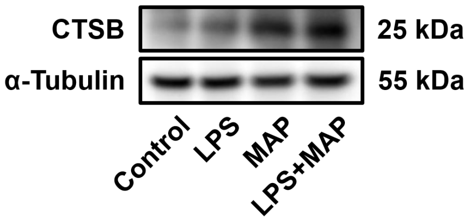

1-CTSB

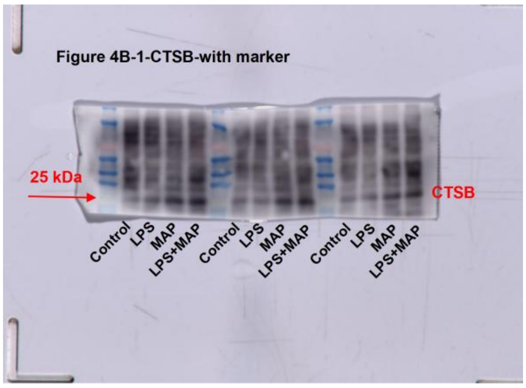

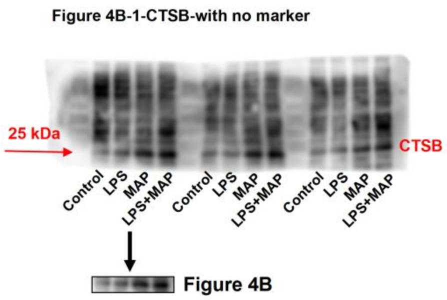

2-α-Tubulin

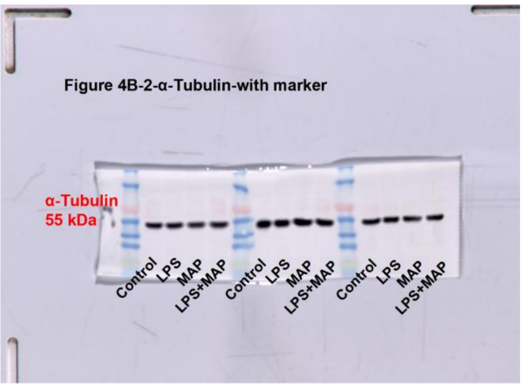

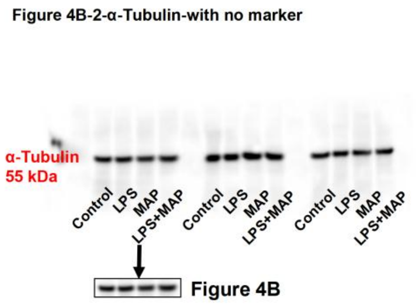

Figure S3. Uncropped Western blot images in Figure 4B.

Figure 5A

(A)

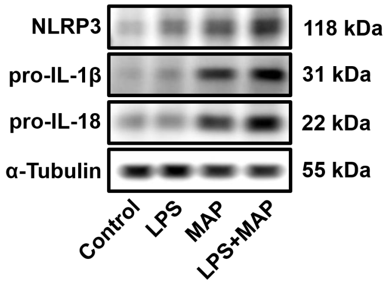

1-NLRP3

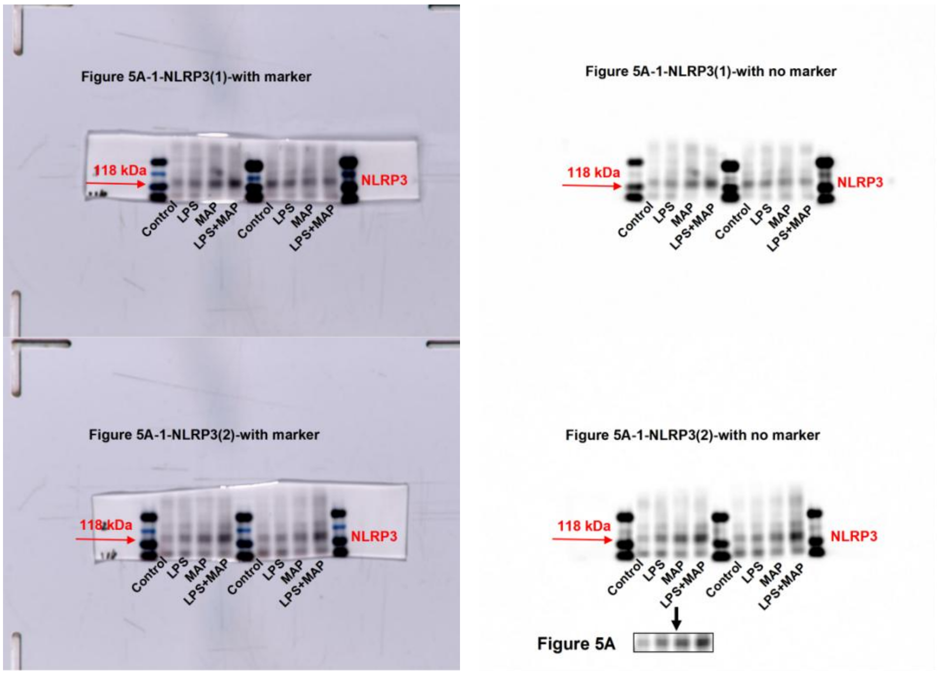

2-pro-IL-1β

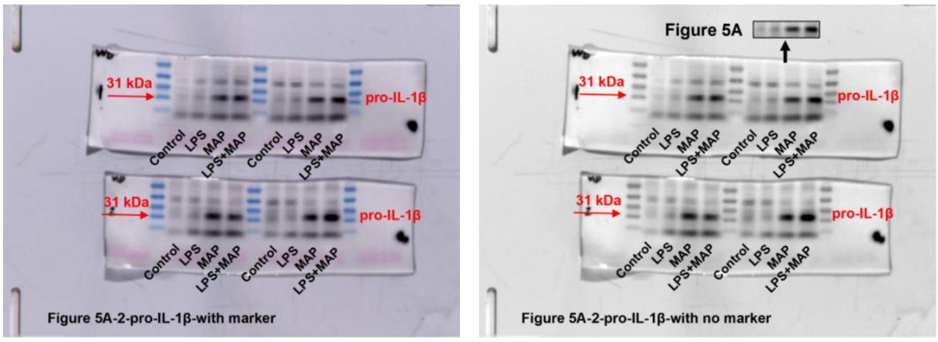

3-α-Tubulin

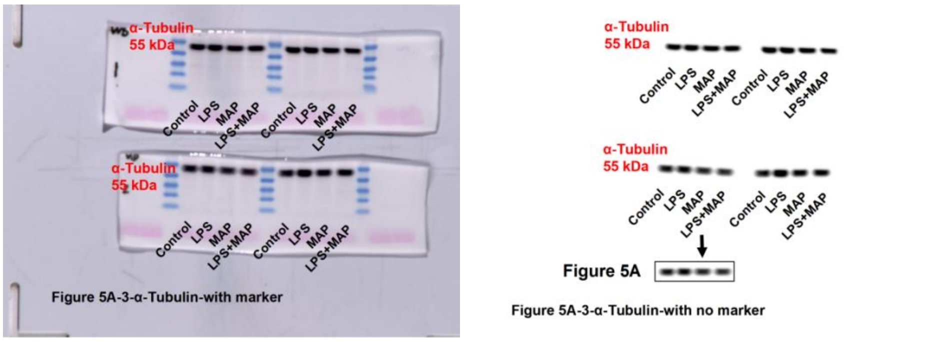

4-pro-IL-18

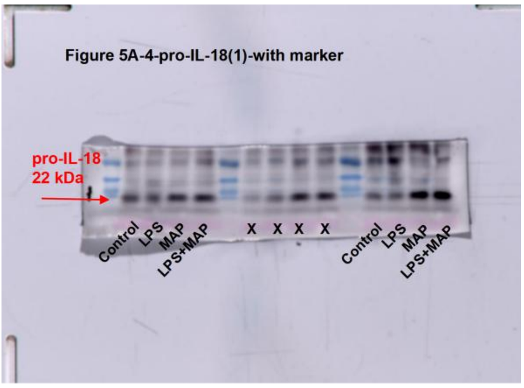

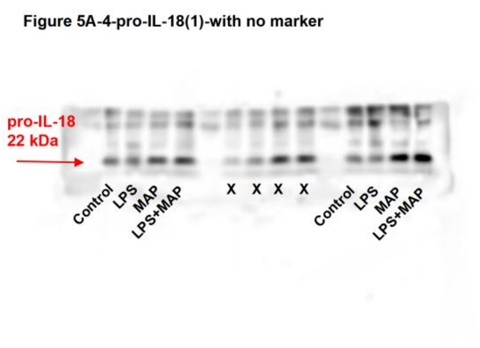

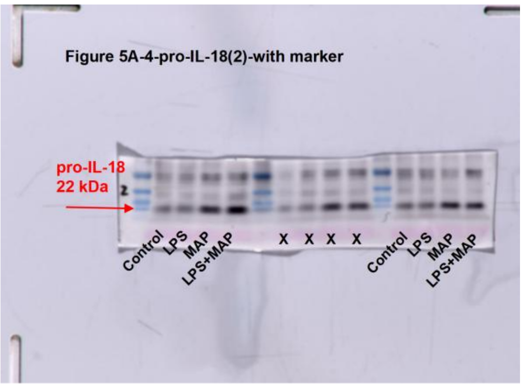

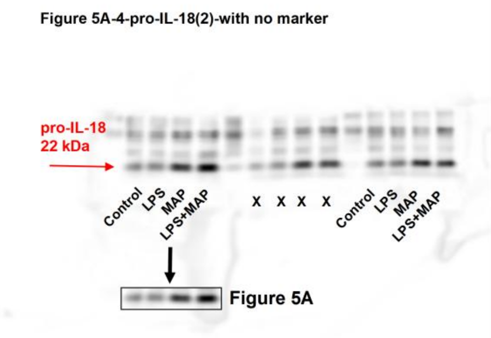

5-α-Tubulin

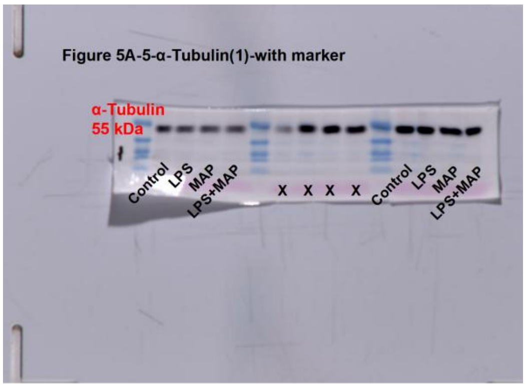

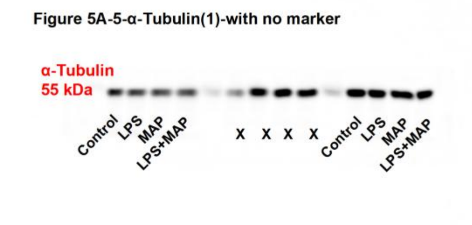

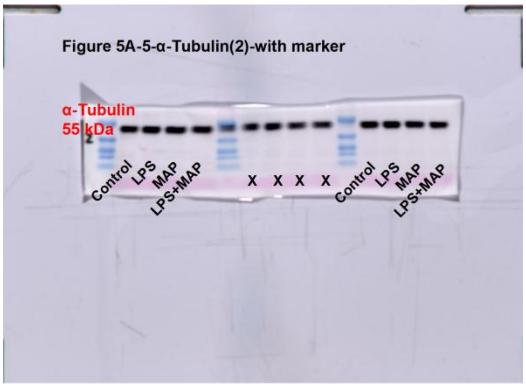

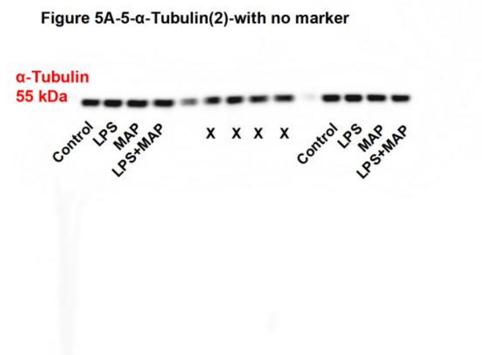

Figure S4. Uncropped Western blot images in Figure 5A.

Figure 5B

(B)

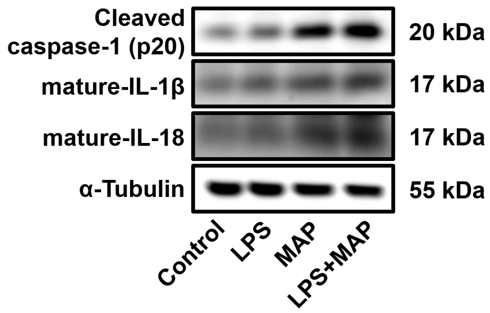

1-p20

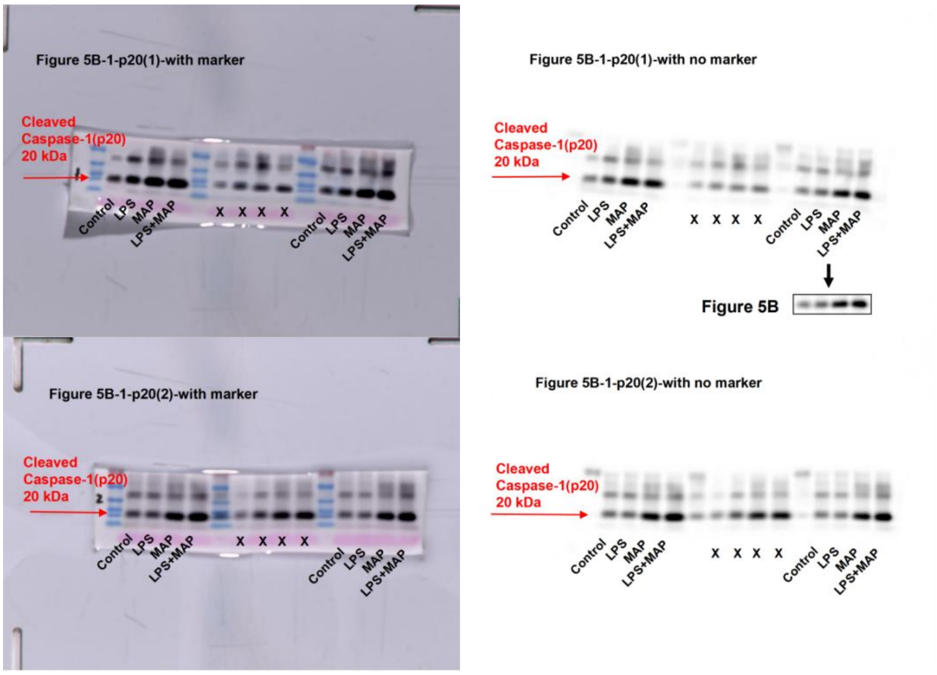

2-mature-IL-18

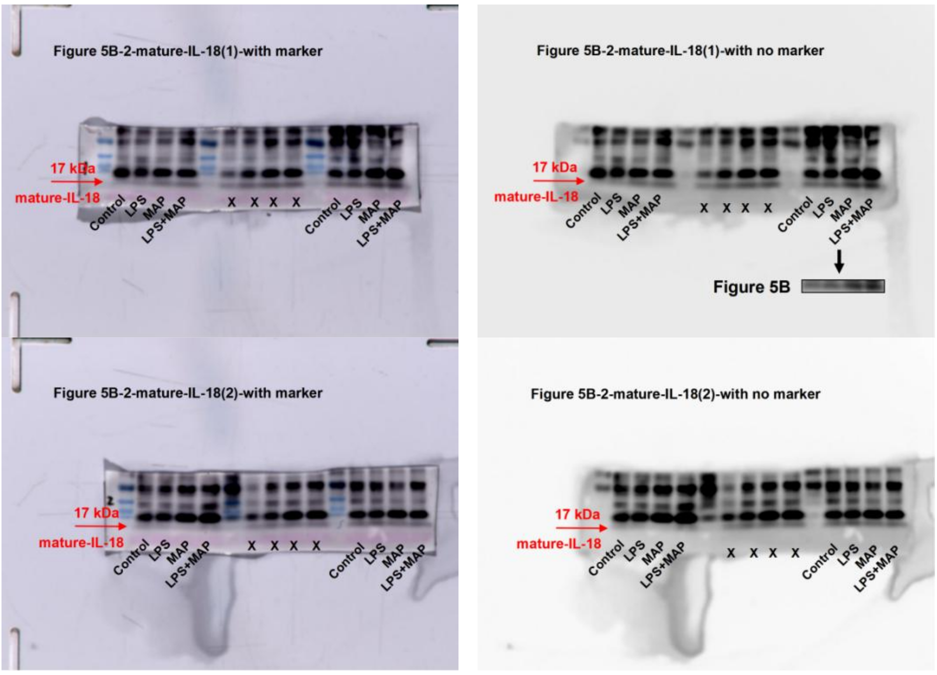

3-α-Tubulin

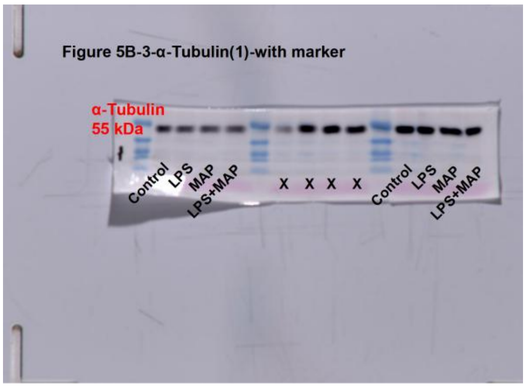

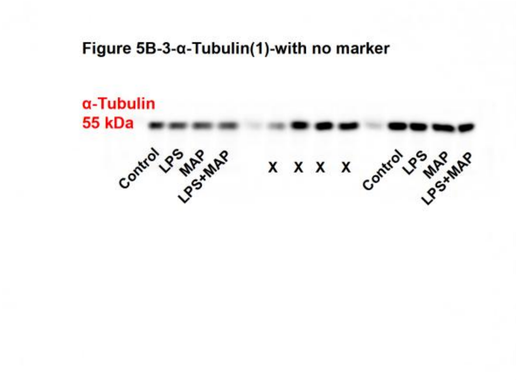

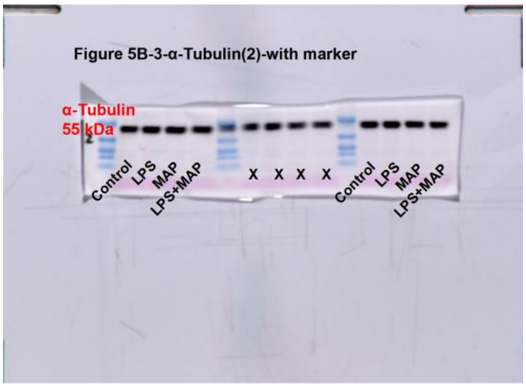

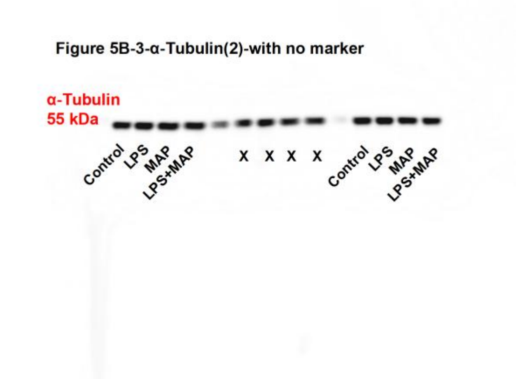

4-mature-IL-1β

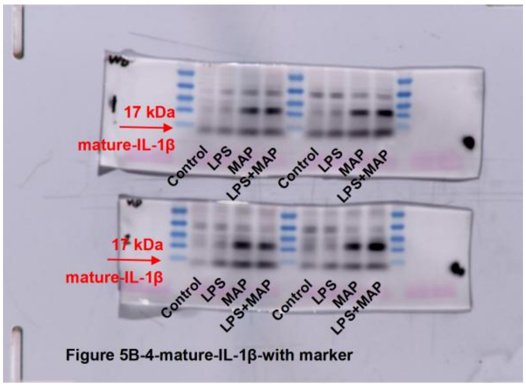

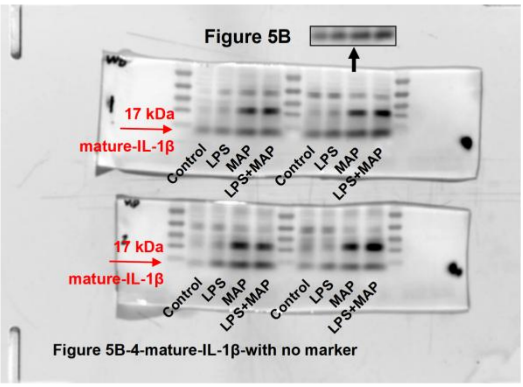

5-α-Tubulin

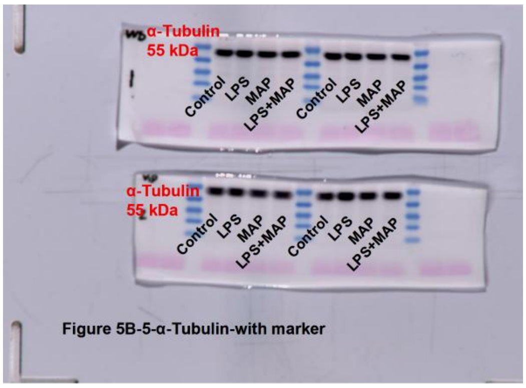

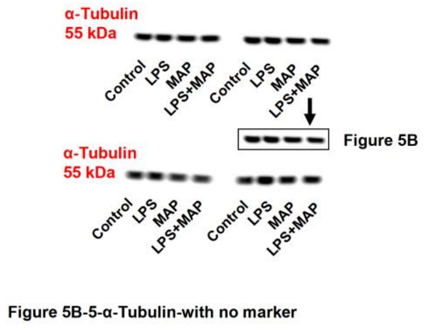

Figure S5. Uncropped Western blot images in Figure 5B.

Figure 7C

(C)

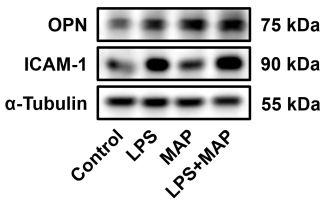

1-OPN

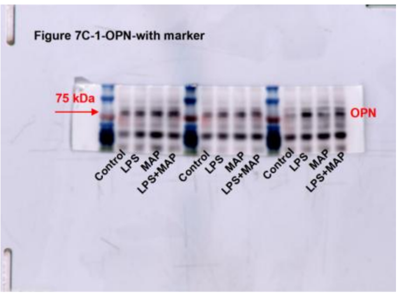

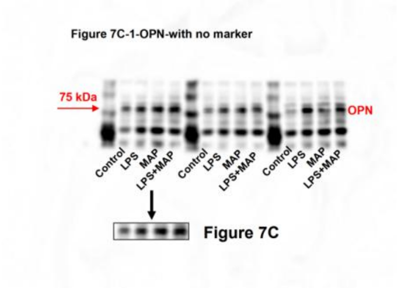

2-α-Tubulin

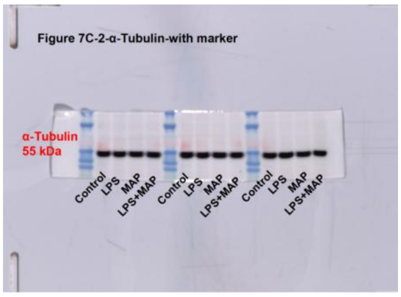

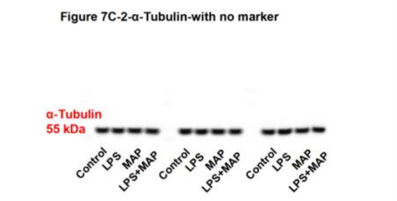

3-ICAM-1

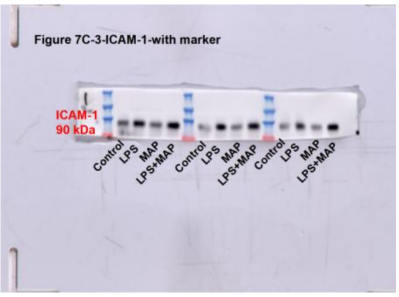

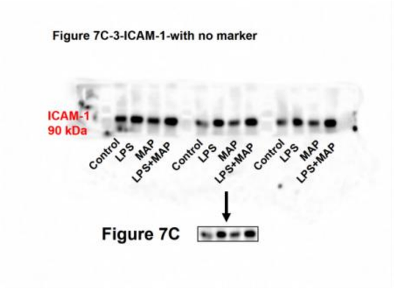

4-α-Tubulin

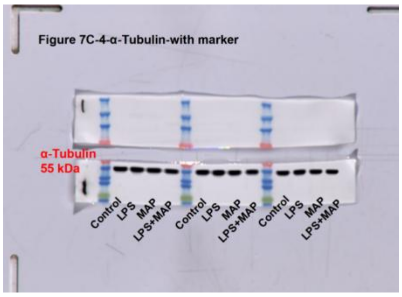

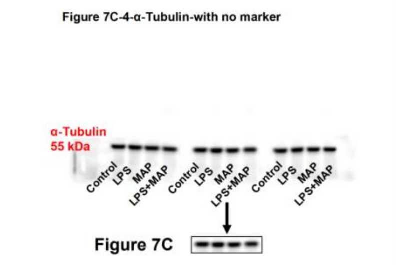

Figure S6. Uncropped Western blot images in Figure 7C.
